# Terpyridine-Grafted Nitrogen-Terminal Endowing Cyanine with Metal-Ion-Regulated Photophysical Properties for Cancer Theranostics

**DOI:** 10.34133/research.0061

**Published:** 2023-02-27

**Authors:** Junfei Zhu, Gang He, Peng-Hang Chen, Yajie Zhang, Yafei Zhang, Shan Lei, Yu Zhang, Meng Li, Peng Huang, Jing Lin

**Affiliations:** Marshall Laboratory of Biomedical Engineering, International Cancer Center, Laboratory of Evolutionary Theranostics (LET), Guangdong Key Laboratory for Biomedical Measurements and Ultrasound Imaging, School of Biomedical Engineering, Shenzhen University Medical School, Shenzhen 518060, China.

## Abstract

Heptamethine cyanines (Cy7) are one of the most important dyes in bioimaging and phototherapy, but they often suffer from poor photostability or limited photothermal conversion efficiency. Here, a facile molecular engineering approach to regulating the photophysical properties of Cy7 by metal ions is demonstrated. By innovatively modifying the nitrogen with functional groups, a novel terpyridine-grafted nitrogen-terminated Cy7 scaffold (denoted as CydtPy) was synthesized and exhibited tunable photophysical properties when chelating with various metal ions (Mn^2+^, Fe^2+^, etc.). In comparison with metal-ion-free PEGylated CydtPy (LET-11), Mn^2+^-chelated LET-11 (namely, LET-11-Mn) exhibited the increased fluorescence emission intensity, and Fe^2+^-chelated LET-11 (namely, LET-11-Fe) showed the enhanced photostability with ~2-fold increase in photothermal conversion efficiency. By simply switching the chelated metal ion species, LET-11-Mn or LET-11-Fe could be used for near-infrared fluorescence imaging, magnetic resonance imaging, or photoacoustic imaging. Furthermore, LET-11-Fe displayed superior synergistic efficacy of photothermal therapy and chemodynamic therapy both in vitro and in vivo. This work not only provides a new strategy for regulating the photophysical properties of cyanine dyes but also establishes a versatile nanoplatform for cancer theranostics.

## Introduction

The nanotheranostics that integrates diagnosis and treatment allows for the imaging and treatment of cancer as well as real-time monitoring of the treatment effect, which has become a trend in precision medicine [1–3]. Compared with the more or less deficiencies in sensitivity, spatial resolution, or penetration depth of a single imaging modality, multimodal imaging strategies help to overcome these limitations and provide complementary and accurate insights into tumor characteristics [4–7]. However, because of the different imaging principles of each imaging system, it is still a great challenge to achieve dual or more modalities imaging based on a single molecular probe [8–10]. In addition, research has demonstrated that monotherapy lacks sufficient potential to completely eliminate tumors and inhibit tumor cell metastasis [11,12]. To date, the explosive growth of synergistic therapeutic strategies has demonstrated “1 + 1 > 2” therapeutic effects [13–15]. Among them, the integrated photothermal therapy (PTT) and chemodynamic therapy (CDT) strategy has recently emerged as a well-liked alternative for antitumor therapy [16]. Kinetic studies elucidate that increasing temperature can validly accelerate the metal-ion-mediated Fenton/Fenton-like reaction and improve the generation rate of hydroxyl radical (•OH) for a better CDT efficacy [17]. Interestingly, •OH produced by CDT fully complies with the requirement of heat shock protein activity inhibition, thereby enhancing the PTT efficacy [18,19]. Therefore, PTT/CDT is a clearly promising synergistic bimodal therapeutic candidate due to the complementarity of PTT and CDT, rather than a simple combination of them [20–24].

Near-infrared (NIR) absorbing heptamethine cyanines (Cy7), such as clinical approval of indocyanine green, have been extensively studied for PTT of cancer [25,26]. Unfortunately, such Cy7 dyes suffer from photobleaching and poor photostability, which seriously hinder their clinical applications because of their short duration time staying within the effective temperature of PTT [27,28]. In addition, their photothermal conversion efficiency (PCE) needs to be further improved to meet tumor ablation requirements under low and safe laser irradiation. Therefore, new strategies have to be proposed to simultaneously improve the photostability and PCE of cyanine dyes. The key factor of enhancing photostability is to impede the photoinduced electron transfer (PET) between the excited fluorophore and reactive oxygen species (ROS) [29]. This PET process results in a large quenching of fluorescence, which favors nonradiative decay processes and promotes heat generation after photon absorption [30]. In fact, the direct structural modification of fluorophores can lead to great change in photostability and PCE. One general approach is to replace the chlorine group with the strong electron-withdrawing unit in meso-position of Cy7 structure for enhancing photostability and PCE [31–33]. So far, there is no report on nitrogen-terminal modification of Cy7 with metal-ion-regulated photostability and PCE. In addition, the combination of Cy7 and metal ions shows great potential for cancer theranostics, especially for multimodal-imaging-guided synergistic PTT/CDT of tumor [34].

**Fig. 1.**
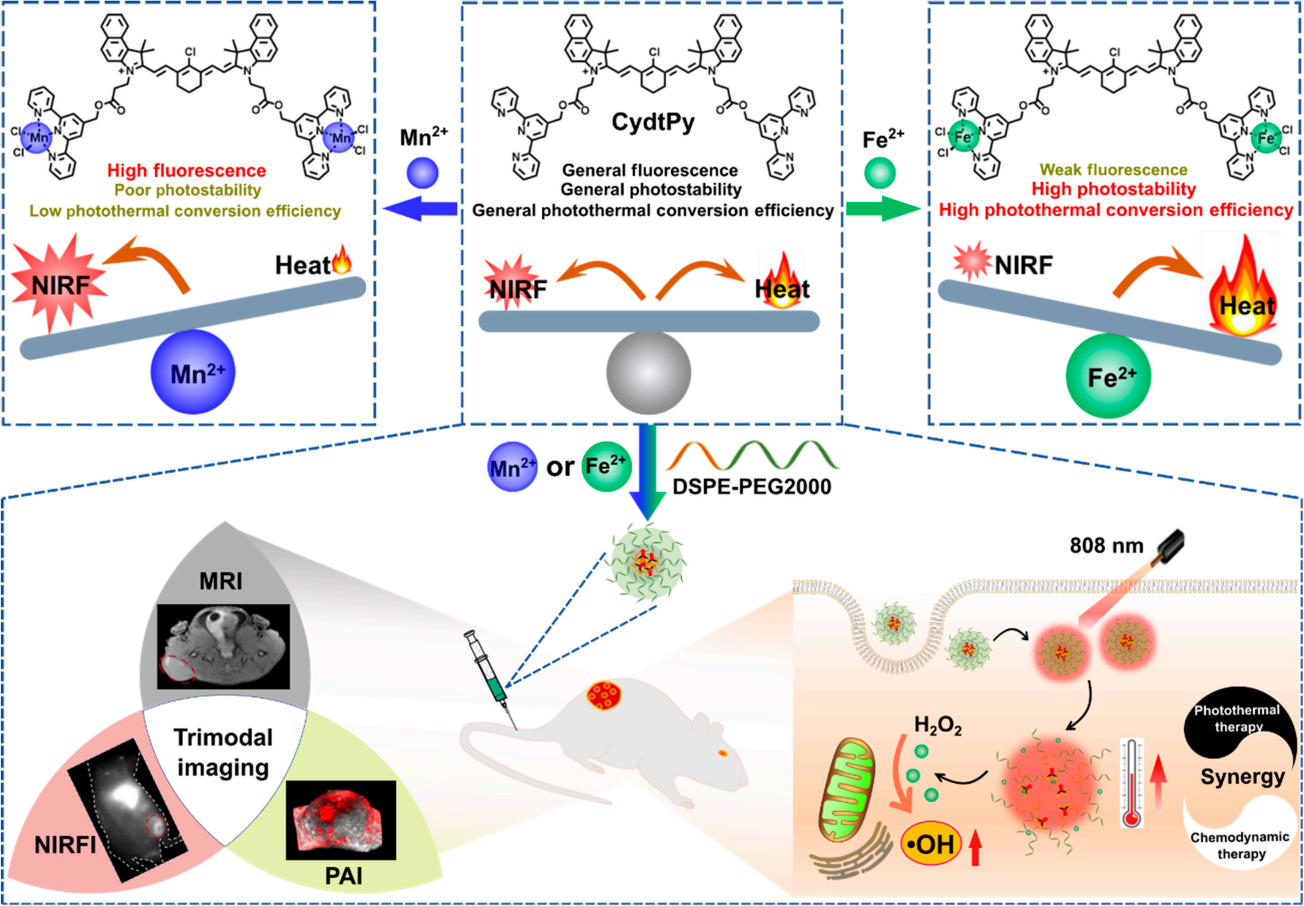
Schematic illustration of terpyridine-grafted nitrogen-terminal endowing cyanine with metal-ion-regulated photophysical properties for multimodal-imaging-guided synergistic PTT/CDT of tumors.

Here, we designed a novel terpyridine-grafted nitrogen-terminated Cy7 scaffold, named as CydtPy, with 2 metal-ion-chelating handles (Fig. 1). When CydtPy was chelated with Mn^2+^, the fluorescence emission of the metal complex was enhanced. However, it displayed fluorescence quenching when chelated with Co^2+^, Cu^2+^, or Fe^2+^. Compared with metal-ion-free PEGylated CydtPy (LET-11), the metal-ion-chelated LET-11 (e.g., LET-11-Fe, LET-11-Co, or LET-11-Cu) demonstrated the higher PCE and enhanced photostability, and LET-11-Mn exhibited the increased fluorescence emission intensity. The as-prepared LET-11-Mn or LET-11-Fe could be used for trimodal imaging, such as NIR fluorescence imaging (NIRFI), magnetic resonance imaging (MRI), and photoacoustic imaging (PAI). Taking advantages of the enhanced photothermal effect of Fe^2+^ and its intrinsic Fenton activity, the LET-11-Fe could be used for synergistic PTT/CDT against tumors. Upon laser irradiation, the photothermal effect of LET-11-Fe could prime the Fe^2+^ release, thus triggering the intracellular Fenton reaction with the abundant generation of •OH. Briefly, the as-prepared nanoplatform could be employed for multimodal-imaging-guided synergistic PTT/CDT of tumors.

## Results

### Metal-ion-regulated photophysical properties

**Fig. 2.**
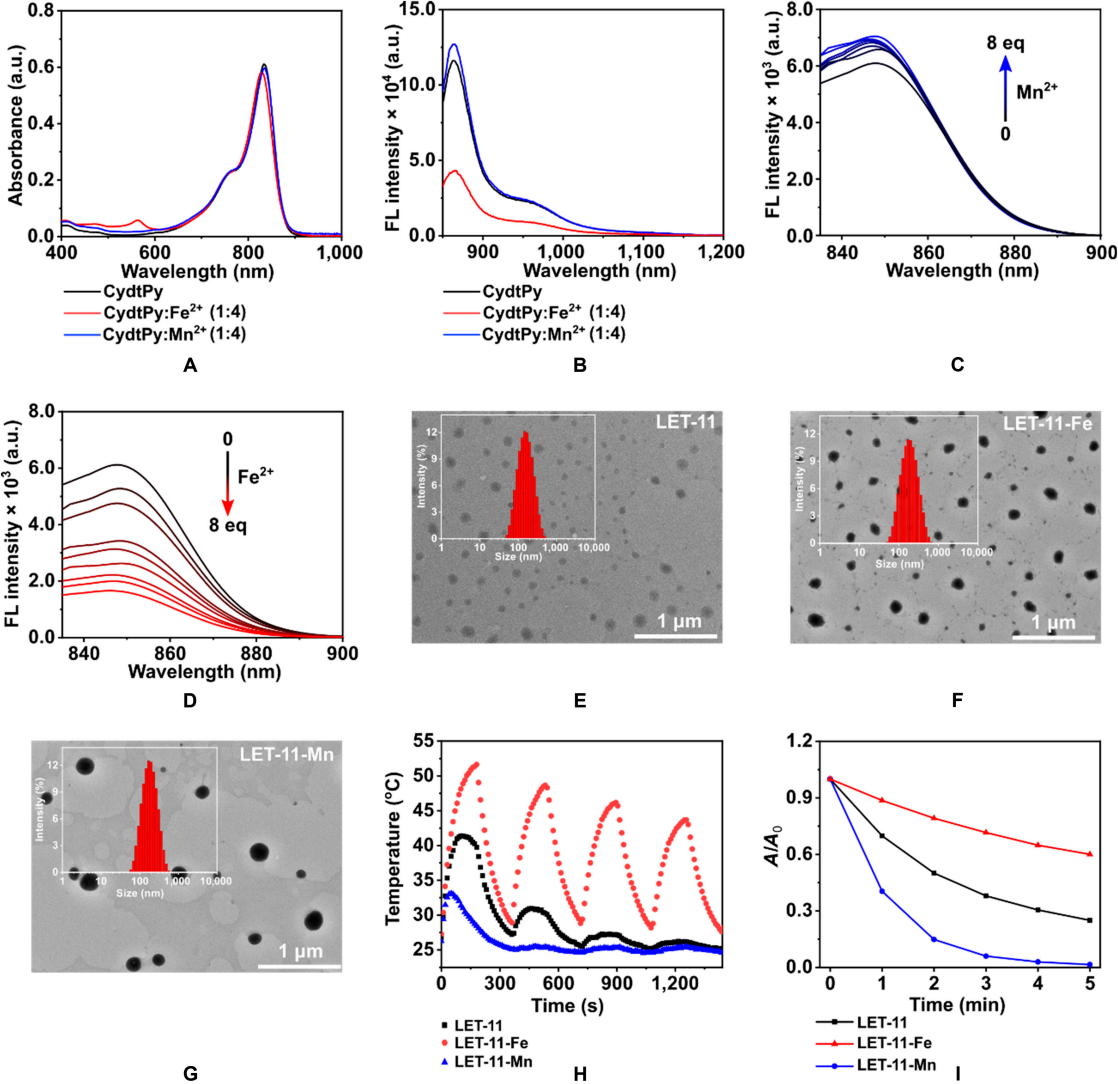
Characterization of as-prepared nanoparticles. (A) UV-vis-NIR absorption and (B) fluorescence spectra of CydtPy, CydtPy:Fe^2+^, and CydtPy:Mn^2+^ in DMSO. Fluorescence spectra of CydtPy upon the addition of (C) Mn^2+^ ions and (D) Fe^2+^ ions (0 to 8 eq). DLS and TEM images of (E) LET-11, (F) LET-11-Fe, and (G) LET-11-Mn. (H) Cyclic photothermal heating and cooling of LET-11, LET-11-Fe, and LET-11-Mn (808 nm, 0.3 W cm^−2^). (I) Photostability of LET-11, LET-11-Fe, and LET-11-Mn under laser irradiation (808 nm, 1 W cm^−2^). a.u., arbitrary units.

The functional cyanine CydtPy was synthesized following the procedure described in Fig. S1. The intermediates and the final products were characterized by nuclear magnetic resonance (^1^H NMR and ^13^C NMR) and mass spectrometry (Figs. S2 to S8). The ability of CydtPy to coordinate with different metal ions (i.e., Mn^2+^, Fe^2+^, Cu^2+^, and Co^2+^) was evaluated in dimethyl sulfoxide (DMSO) solution by observing the changes in the absorption and fluorescence spectra. The absorption spectra (Fig. 2A and Fig. S9A) of CydtPy showed no observable change, but the fluorescence spectra of CydtPy obviously changed (Fig. 2B and Fig. S9B) with the addition of divalent metal ions (i.e., Mn^2+^, Fe^2+^, Cu^2+^, and Co^2+^), indicating the formation of metal complexes. Among them, the fluorescence intensity of CydtPy slightly enhanced with the concentration increase in chelating Mn^2+^ (Fig. 2C) and dramatically decreased as the concentration increase in Fe^2+^, Cu^2+^ and Co^2+^ (Fig. 2D and Fig. S9B). This phenomenon could be attributed to the chelation-enhanced fluorescence PET mechanism between CydtPy and metal ions [35–37]. To improve the biocompatibility of CydtPy for in vivo biomedical application, the CydtPy was encapsulated with amphiphilic 1,2-distearoyl-*sn*-glycero-3-phosphoethanolamine-*N*-[methoxy(polyethyleneglycol)2000] (DSPE-PEG2000) to form LET-11 micellar nanoparticles. The metal-ion-chelated LET-11 was prepared by the same method after CydtPy was chelated with different metal ions. All as-prepared nanoparticles exhibited uniform sphere-like morphology and narrow size distribution, which were confirmed by dynamic light scattering (DLS) measurement (hydrodynamic diameter of about 110 nm) and transmission electron microscope (TEM) observation (Fig. 2E to G and Fig. S10). Furthermore, all these nanoparticles displayed good stability even after 1-week storage (Fig. S11). The fluorescence intensity of above nanoparticles was weakened after DSPE-PEG2000 encapsulation, but their fluorescence spectrum change trends caused by chelated metal ions were same as before encapsulation (Fig. S12). According to Jablonski energy diagram, the light energy absorbed by chromophores decay through 3 transition pathways, namely, fluorescence emission (radiative relaxation process), photochemical reactions (ROS generation), and heat (nonradiative relaxation process) [38]. Therefore, the fluorescence change caused by chelated metal ions may affect the PCE of CydtPy. We further investigated the photothermal effect of this series of nanoparticles under 808-nm laser irradiation. As shown in Fig. 2H, the temperature of LET-11-Fe solution raised to 51.6 °C during 3-min irradiation at 0.3 W cm^−2^ and was much higher than that of LET-11 solution (41.2 °C). The PCE of LET-11-Fe was determined to be 21.9 %, which was about 2-fold higher than that of LET-11 (11.7%) (Fig. S13). In contrast, the temperature of LET-11-Mn solution (33.1 °C) was much lower than that of LET-11 solution, and its PCE (6.9%) was about 2-fold lower than that of LET-11. Notably, LET-11-Fe exhibited good photothermal efficiency compared with the almost zero photothermal efficiency of LET-11 in the 4th cycle, indicating its potential for photothermal applications (Fig. 2H). Antiphotobleaching experimental results showed that even with high-power laser irradiation (808 nm, 1 W cm^−2^) for 5 min, LET-11-Fe was only degraded by about 40%, which is much lower than 75% of LET-11 and 99% of LET-11-Mn (Fig. 2I). In addition, other divalent metal ions, such as Co^2+^ and Cu^2+^, have similar regulating functions as Fe^2+^ and can also improve the photostability and PCE of CydtPy (Figs. S14 and S15). The ROS generation during reactions between molecular oxygen and fluorophore triplets are the main cause of the photobleaching of most organic fluorophores [39]. Evidence is described that Fe^2+^, Cu^2+^, and Co^2+^ allowed d-d transitions, leading to the quenching of the singlet state of the cyanine dyes by an electron-exchange mechanism, thus enhancing the photostability of CydtPy [40,41]. In contrast, Mn^2+^, due to its paramagnetic character, promotes intersystem crossing to the triplet state of the cyanine dyes, leading to the accelerated photobleaching [42]. Therefore, it can be concluded that the photostability and PCE of CydtPy can be regulated by changing the type of chelated metal ions.

### Metal-ion-regulated trimodality imaging in vitro

**Fig. 3.**
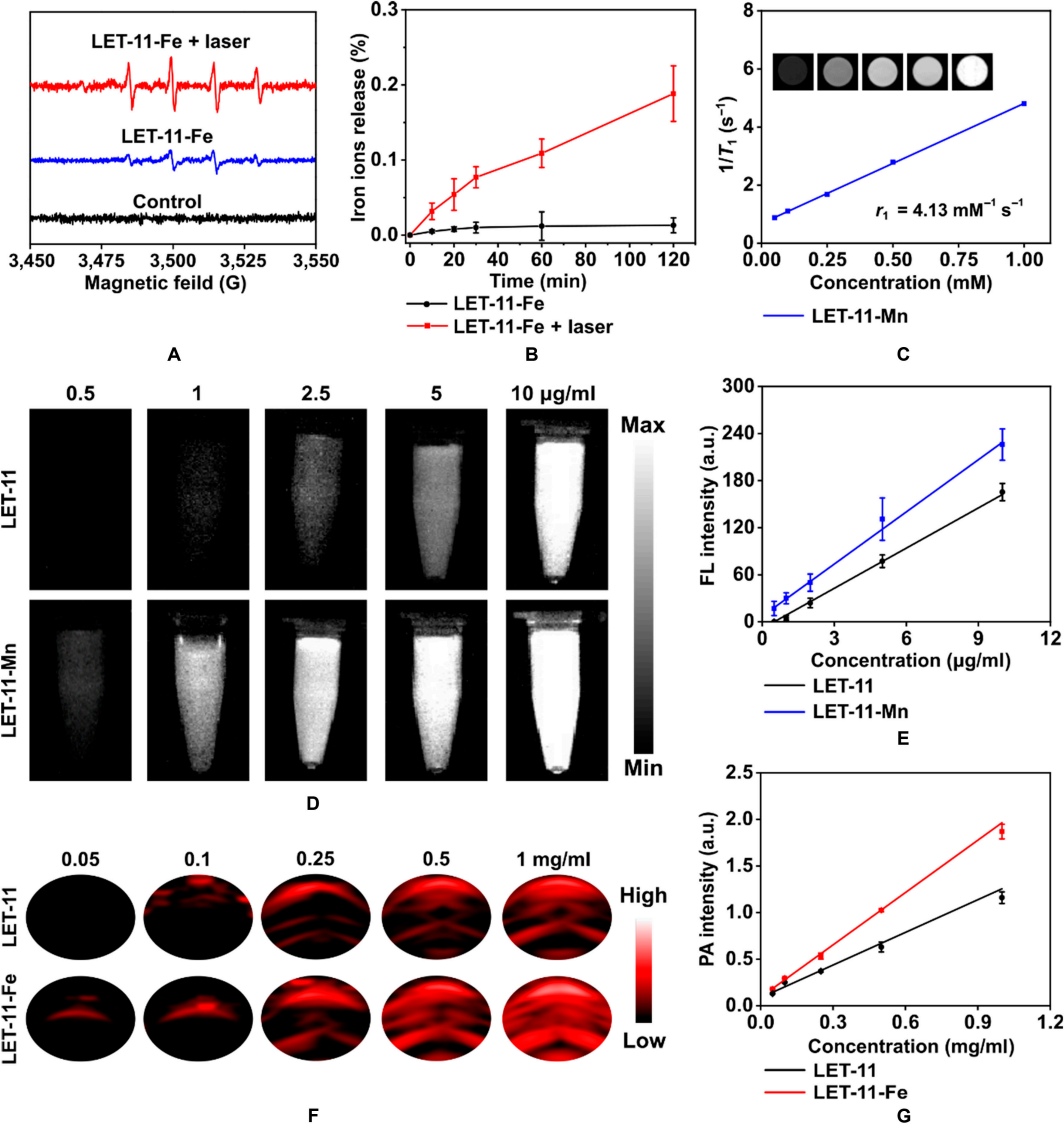
Trimodality imaging in vitro. (A) ESR spectra of LET-11-Fe solution incubated with H_2_O_2_ and DMPO with or without laser irradiation. (B) Release profiles of iron ions from LET-11-Fe with or without laser irradiation (*n* = 3). (C) Plots of *T*_1_^−1^ versus Mn concentration for LET-11-Mn and *T*_1_-weighted MRI images (inset). (D) Fluorescence images of LET-11 and LET-11-Mn at different concentrations (excitation, 808 nm; 1,000-nm long-pass emission filter). (E) Fluorescence intensity as a function of LET-11 and LET-11-Mn at different concentrations. (F) PA images of LET-11 and LET-11-Fe under excitation at 835 nm at different concentrations. (G) PA_835_ intensity as a function of LET-11 and LET-11-Fe at different concentrations.

It was well known that Mn^2+^, Fe^2+^, Co^2+^, and Cu^2+^ have Fenton’s catalytic activity [43]. Hence, methylene blue (MB) oxidation assay was conducted in the presence of hydrogen dioxide (H_2_O_2_) and metal-ion-chelated LET-11 to detect the formation of •OH. Notably, laser irradiation markedly increased the degradation of MB (Fig. S16), indicating more •OH generation and achieving higher catalytic efficiency. Furthermore, taking LET-11-Fe as an example, electron spin resonance spectroscopy (ESR) experiments obtained the similar results as MB oxidation using 5,5-dimethyl-1-pyrroline-N-oxide (DMPO) as the •OH trapping agent (Fig. 3A). The underlying mechanism of light-enhanced Fenton reaction of LET-11-Fe can be explained from 2 aspects. On the one hand, the heat generated from LET-11-Fe with 808-nm laser irradiation can effectively increase the speed of Fenton reaction [17]. On the other hand, LET-11-Fe was disintegrated under 808-nm laser irradiation, resulting in the exposure and release of Fe^2+^, which promoted the occurrence of the Fenton reaction. The inductively coupled plasma mass spectrometry analysis of iron element (Fig. 3B) and the changes of hydrodynamic diameter and morphology (Fig. S17) before and after laser irradiation further confirmed this hypothesis. All the above results suggested that LET-11-Fe has the potential for synergistic PTT/CDT of tumors. Next, we investigated the multimodal imaging performances of metal-ion-chelated LET-11 in vitro. Mn^2+^ not only enhanced the fluorescence emission of LET-11 but also should be an ideal longitudinal (*T*_1_)-MRI contrast agent due to its relatively high electronic spin and fast water exchange rates [44]. The longitudinal relaxivity (*r*_1_) of LET-11-Mn was calculated to be 4.13 mM^−1^ s^−1^ by plotting the inverse relaxation time against the Mn concentration (Fig. 3C). The NIRFI images of LET-11 or LET-11-Mn were determined in the concentrations range of 0.5 to 10 μg ml^−1^ and exhibited a good linear correlation (Fig. 3D and E). Notably, the imaging sensitivity of LET-11 was enhanced after chelating Mn^2+^, which is consistent with the results of fluorescence spectra (Fig. 2B and Fig. S12). Meanwhile, the PAI performance of LET-11 after Fe^2+^ chelating was investigated. As presented in Fig. 3F and G, the PA signal at 835 nm (PA_835_) of LET-11-Fe exhibited a linear increase in the range of 0.05 to 1 mg ml^−1^, and its amplitude was slightly higher than that of LET-11 at each concentration, which was attributed to enhanced photothermal transfer caused by Fe^2+^. These data demonstrated that LET-11 has the great potential capability for trimodality imaging (NIRFI/MRI/PAI)-guided synergistic PTT/CDT by simply switching the chelated metal ion species.

### Synergistic PTT/CDT on tumor cells

**Fig. 4.**
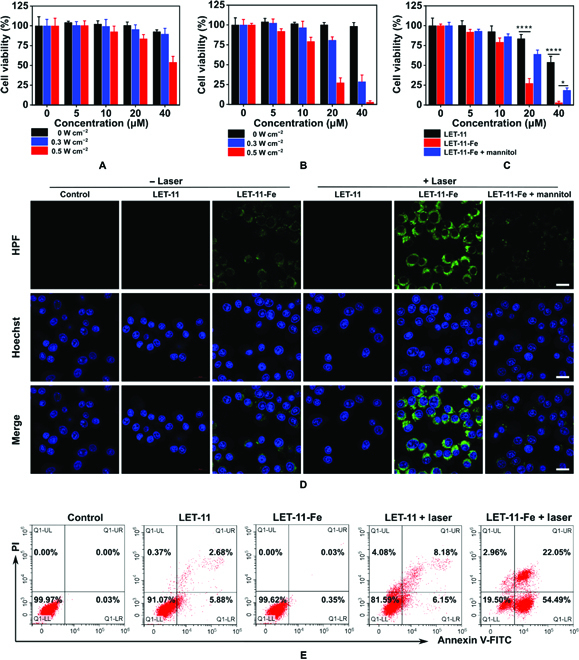
Synergistic PTT/CDT antitumor effect of LET-11-Fe in vitro. Cell viabilities of 4T1 cells incubated with different concentrations of (A) LET-11 or (B) LET-11-Fe with or without 808-nm laser irradiation (0, 0.3, and 0.5 W cm^−2^; *n* = 5). (C) Cell viability of 4T1 cells with different treatments under laser irradiation (808 nm, 0.5 W cm^−2^; *n* = 5). *P < 0.05 and ****P < 0.0001, analyzed by 2-sided Student’s *t* test. (D) HPF-stained fluorescence images of 4T1 cells with different treatments. Scale bars, 20 μm. The irradiation density was 0.3 W cm^–2^ at 808 nm, and the irradiation time was 10 min. (E) Flow cytometry analysis of 4T1 cells apoptosis induced by different treatments under laser irradiation (808 nm, 0.3 W cm^−2^ for 10 min). FITC, fluorescein isothiocyanate.

After confirming the photothermal effect and Fenton’s catalytic activity of LET-11-Fe, its therapeutic efficiency was evaluated by 3-(4,5-dimethylthiazol-2-yl)-2,5-diphenyltetrazolium bromide (MTT) assay on mouse breast (4T1) cancer cells. LET-11 and LET-11-Fe caused negligible toxicity to 4T1 cells even with the concentration up to 40 μM (Fig. 4A and B), indicating the good cytocompatibility. Note that LET-11 showed clear cytotoxicity only at high concentration with high laser irradiation (40 μM, 0.5 W cm^−2^), with a cell viability of 53.8 ± 7.5%, indicating its weak photothermal effect (Fig. 4A). In comparison, LET-11-Fe exhibited more cytotoxic than LET-11 even at low laser irradiation (0.3 W cm^−2^) (Fig. 4B) due to the synergistic effect of PTT/CDT. Concretely, at the concentration of 40 μM, the cell viability of LET-11-Fe was 28.6 ± 8.3% with a laser density of 0.3 W cm^−2^, which was 3.1-fold lower than that of LET-11 (89.5 ± 7.3%), showing its significant light-triggered antitumor efficacy. To gain insight into the CDT efficacy of LET-11-Fe, mannitol was used as the •OH scavenger [45]. As shown in Fig. 4C, under laser irradiation, the LET-11-Fe with mannitol-treated group exhibited lower cellular destruction than LET-11-Fe group because of the •OH scavenger. Such phenomena were also confirmed by calcein-acetoxymethyl ester/propidium iodide (PI) costaining images (Fig. S18). The generation of •OH was further confirmed by hydroxyphenyl fluorescein (HPF). Compared to Fe^2+^-free group, LET-11-Fe-treated cells showed weak green fluorescence owing to weak CDT effect, while the strong green fluorescence was observed in LET-11-Fe + laser group, indicating that laser irradiation can obviously increase the production efficiency of •OH (Fig. 4D) as result of more light-induced release of Fe^2+^ for Fenton reaction. Next, annexin V-fluorescein isothiocyanate/PI assay was used to distinguish viable cells and dead cells at different phases by flow cytometry, which were identified as viable, early apoptotic, late apoptotic, and necrotic cells, respectively (Fig. 4E). For the cells treated with LET-11-Fe + laser, the cell mortality rate significantly increased to 80.5% (54.5% for early apoptosis, 22.1% for later apoptosis, and 3.0% for necrosis), which is much higher than that of LET-11 + laser (19.4%) or LET-11-Fe (0.4%). These results further suggest that synergetic PTT/CDT induced more cell apoptosis than single PTT or CDT alone.

### Synergistic PTT/CDT effect on cell migration

**Fig. 5.**
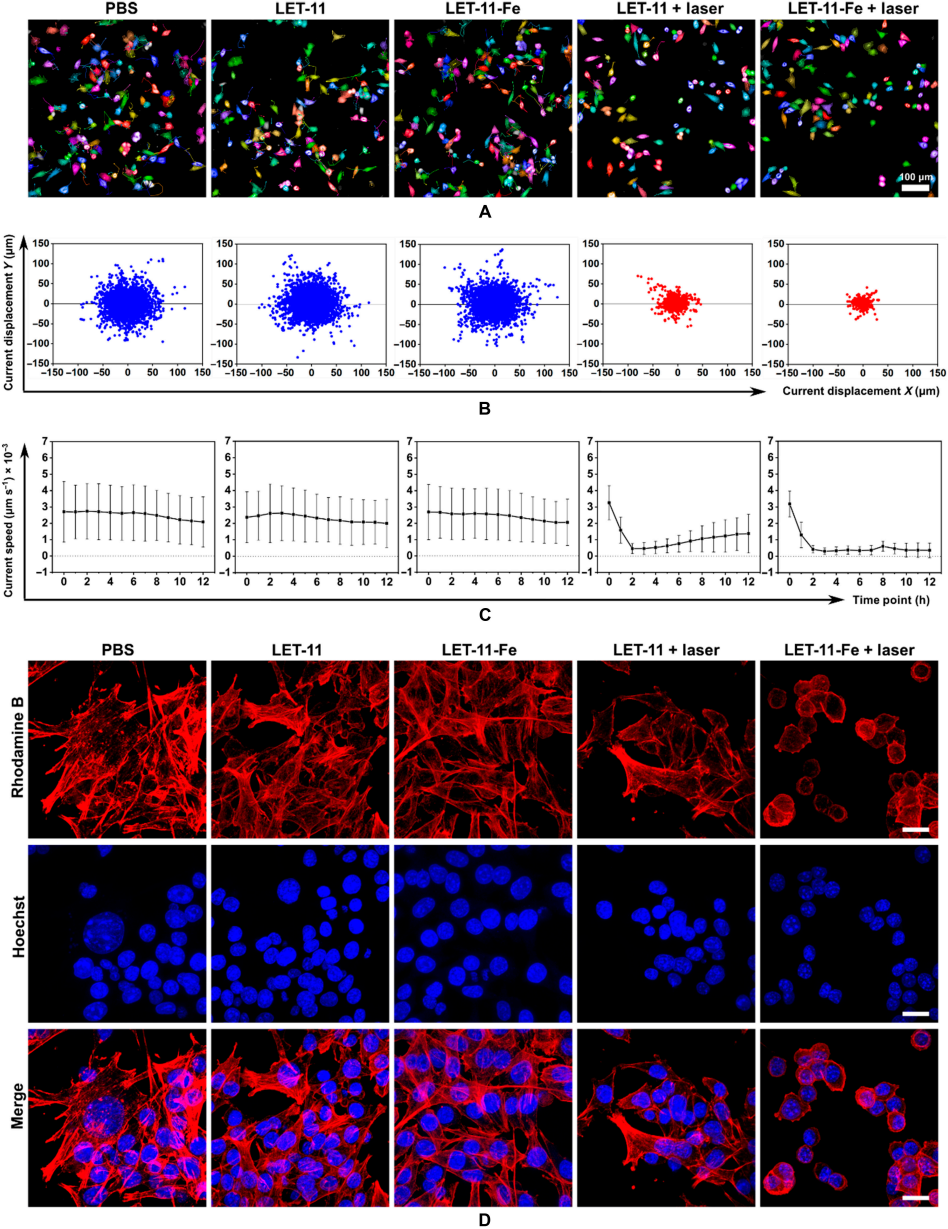
Effect of LET-11-Fe on cell migration. (A) Induction of 4T1 cells migration after indicated treatments. Images are snapshots from videos generated by the Harmony software showing digital phase contrast images with the segmentation mask and the cell track overlaid. Scale bar, 100 μm. (B) Current displacement of 4T1 cells treated with different conditions (*n* = 3 wells). (C) Mean speed over all cells at different time points after indicated treatments (*n* = 3 wells). (D) Confocal fluorescence images of 4T1 cells after various treatments. Scale bars, 20 μm. F-actin was stained with phalloidin (red), and nuclei was stained with Hoechst 33342 (blue). The irradiation density was 0.3 W cm^−2^ at 808 nm, and the irradiation time was 10 min.

The synergistic therapeutic effect of LET-11-Fe under laser irradiation was further examined on cancer cell migration using high-content analysis system [46]. As revealed in the images of movement trajectory at 12 h (Fig. 5A and B), both LET-11 and LET-11-Fe with laser-irradiation-treated groups showed the less or almost no cell migrations as indicated by shorter cell tracks than laser-free groups. Meanwhile, their movement speed of laser-treated cells was decreased (Fig. 5C). Notably, LET-11-Fe + laser group got shorter cell tracks and lower movement speed than LET-11 + laser group. It is worth noting that because of the weak photothermal effect of LET-11, the movement speed in LET-11 + laser-treated cells would gradually increase, while movement speed of LET-11-Fe + laser-treated cells was almost zero and remained unchanged, indicating good synergistic antitumor effect of PTT/CDT after chelating Fe^2+^. The migration of cells is mainly driven by their actin cytoskeleton, and we further stained F-actin with phalloidin to observe the distribution of cytoskeletal filaments [47,48]. As displayed in Fig. 5D, F-actin filaments were dramatically decreased after LET-11 or LET-11-Fe under laser irradiation, further indicating impaired cytoskeletal reorganization and protrusion formation, as well as weakened cell polarization. LET-11-Fe + laser group received more serious destruction of F-actin filaments with the formation of a rounded morphology from a stretched shape compared to LET-11 + laser group, which was consistent with cell migration results. All these experiments demonstrated that light-induced LET-11-Fe interfered with the functional role of the cytoskeleton in cell motility via synergetic PTT/CDT effect.

### Metal-ion-regulated trimodality imaging in vivo

**Fig. 6.**
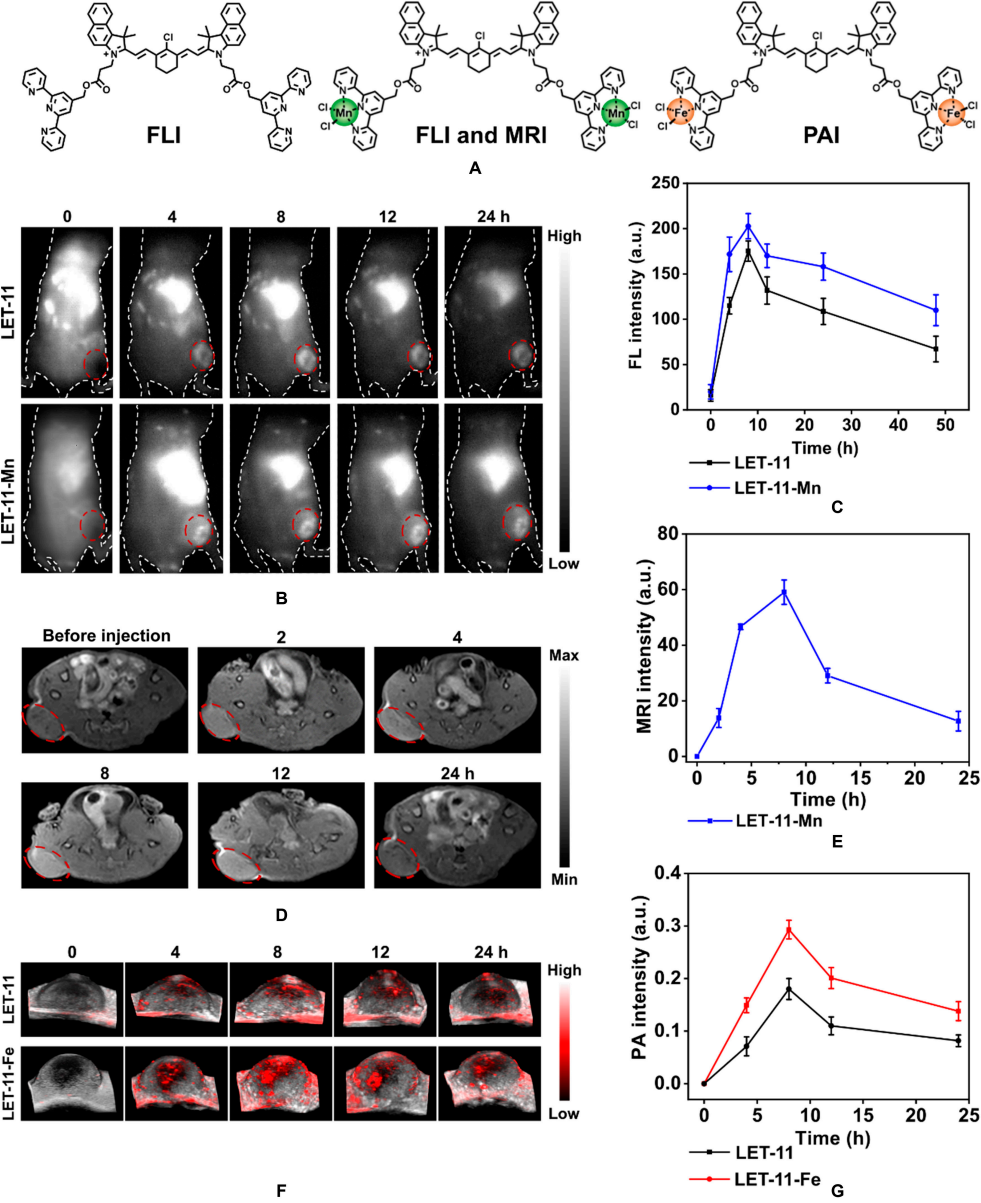
Trimodality imaging in vivo. (A) Schematic representation of molecular structures for different modality imaging. (B) NIRF images of 4T1-tumor-bearing nude mice after intravenous injection of LET-11 or LET-11-Mn. The red circle denotes the tumor site (excitation, 808 nm; 1,000-nm long-pass emission filter). (C) Quantificational analysis of fluorescence intensity in tumor sites at different time points (*n* = 3). (D) In vivo *T*_1_-weighted axial MR images of mice before and after injection of LET-11-Mn. The red circle denotes the tumor site. (E) Quantificational analysis of MR intensity at different time points (*n* = 3). (F) Time-dependent 3-dimensional-rendered PA images of tumor sites after intravenous injection of LET-11 or LET-11-Fe. (G) Quantification of PA intensities of tumors as a function of postinjection time (*n* = 3).

Next, we conducted a comparative study of the trimodality imaging (NIRFI, MRI, and PAI) capabilities of LET-11 after chelating Mn^2+^ and Fe^2+^ in vivo using 4T1-tumor-bearing mice (Balb/c) model, respectively (Fig. 6A). After tail vein injection of LET-11 or LET-11-Mn, the NIRF signal of tumor region was visible at 4 h, reached a plateau at 8 h, and maintained for more than 24 h (Fig. 6B). Noteworthily, the NIRF intensity of LET-11-Mn-treated mice was 1.2-fold higher than that of LET-11-treated mice (Fig. 6C). This should be attributed to the fluorescence signal enhancement by Mn^2+^ chelation as shown in Fig. 2B and Fig. S12. For a more accurate comparison of their fluorescence intensities, mice were sacrificed, and ex vivo NIRFI was performed on isolated organs (heart, liver, spleen, lung, kidney, and tumor) at 48-h postinjection of LET-11 or LET-11-Mn (Fig. S19). Notably, the fluorescence intensity of LET-11-Mn-treated tumor was stronger than that of LET-11. Depending on the MRI function of Mn^2+^, we further performed in vivo *T*_1_-weighted MRI of LET-11-Mn. The MR signal of tumor area increased over time, and a remarkable *T*_1_ contrast between the tumor area and surrounding normal tissue was observed at 8-h postinjection (Fig. 6D and E). Finally, we compared the PAI capability of LET-11 and LET-11-Fe. As presented in Fig. 6F, the PA images of the tumor area were recorded over time. Quantitative analysis showed that the PA intensity reached a maximum at 8 h (Fig. 6G). The PA intensity of LET-11-Fe group is 1.6 times higher than that of LET-11 group, due to the better PCE of LET-11-Fe. The above trimodality imaging results provide a meaningful guidance for in vivo therapy.

### Antitumor efficacy of LET-11-Fe in vivo

**Fig. 7.**
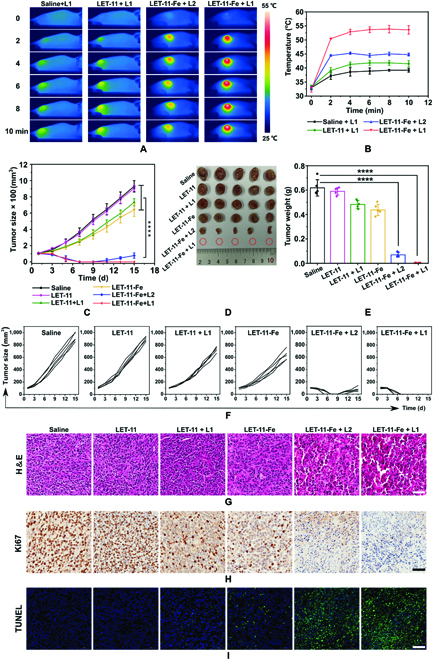
Synergistic PTT/CDT antitumor effect of LET-11-Fe in vivo. (A) Thermal images of 4T1-tumor-bearing mice under 808-nm laser irradiation for 10 min (L1 indicates 0.8 W cm^−2^ and L2 indicates 0.4 W cm^−2^). (B) Mean temperatures of tumors as a function of irradiation time. (C) Tumor growth curves, (D) representative photographs of dissected tumors, and (E) tumor weights after various treatments (*n* = 5). (F) Tumor growth curves of 4T1-tumor-bearing mice after different treatments. (G) H&E, (H) Ki67, and (I) TUNEL staining of tumor tissues. Scale bars, 50 μm. The data are presented as means ± SD, ****P < 0.0001, analyzed by 2-sided Student’s *t* test.

Inspired by the outstanding antitumor efficacy of LET-11-Fe in vitro, the in vivo therapeutic effect was investigated on 4T1-tumor-bearing mice. On the basis of the trimodality imaging results, 808-nm laser irradiation for 10 min was carried out at 8-h postinjection of LET-11 or LET-11-Fe. As shown in Fig. 7A, the tumor temperature of all groups reached a plateau at 4 min and remained constant during the irradiation. The maxima tumor temperature of LET-11-Fe-treated mice was 53.8 and 45.2 °C with laser power density at 0.8 and 0.4 W cm^−2^, respectively. Notably, the maximal tumor temperature of LET-11-Fe group was 12 and 14.6 °C higher than that of LET-11 and saline groups at 0.8 W cm^−2^, respectively (Fig. 7B), as a result of higher PCE of LET-11 after chelating Fe^2+^. To quantitatively evaluate the therapeutic efficacy of different treatments, the tumor volumes were recorded and plotted every 2 d for 15 d after laser irradiation (Fig. 7C). Without laser irradiation, the tumor growth rate of LET-11-Fe-treated mice was slightly reduced compared with the mice treated with saline and LET-11, which should be attributed to CDT effect. Limited by low photothermal effect, LET-11 only slightly slowed tumor growth even at a high laser power density of 0.8 W cm^−2^. It should be noted that the tumor growth was significantly inhibited by LET-11-Fe at a laser power density of 0.4 W cm^−2^ in the first 9 d, indicating that even mild hypothermia can effectively enhance the CDT effect. Excitingly, under the laser irradiation (0.8 W cm^−2^), mice treated with LET-11-Fe completely eradicated tumors without recurrence after a single treatment throughout the experimental period (Fig. 7D to F). These results demonstrated the antitumor efficacy of synergetic PTT and CDT. In addition, no significant body weight changes were found in each group during the treatment process (Fig. S20). Furthermore, hematoxylin and eosin (H&E) staining demonstrated that extensive nuclear shrinkage and disappearance were observed in the tumor slices from LET-11-Fe-treated mice with laser irradiation, which was more severe than that of LET-11 + L1- and LET-11-Fe-treated mice, evidencing its successful destruction of tumor (Fig. 7G). Meanwhile, terminal-deoxynucleotidyl-transferase-mediated deoxyuridine triphosphate nick end labeling (TUNEL) and Ki67-positive immunohistochemical staining also revealed the highest apoptotic levels (green spots) and the lowest proliferation (brown spots) from the synergetic PTT/CDT group (Fig. 7H and I). More importantly, hematology analysis and H&E staining images of main organs (heart, liver, spleen, lung, and kidneys) in LET-11-Fe + laser group showed no physiological abnormalities (Figs. S21 and S22). Collectively, these results verified that LET-11-Fe has good biocompatibility and remarkable capacity for cancer theranostics.

## Discussion

In summary, we successfully synthesized a CydtPy whose photophysical properties can be regulated by intramolecular chelation of metal ions. The LET-11-Mn exhibited the increased fluorescence emission intensity, and LET-11-Fe showed substantially enhanced photostability with ~2-fold increase in PCE. The LET-11-Mn and LET-11-Fe were used as imaging contrast agents, allowing real-time monitoring of delivery process. The “all-in-one” theranostic system based on LET-11-Fe displayed superior synergistic efficacy of PTT and CDT both in vitro and in vivo. Especially, after a single-dose injection, the orthotopic 4T1 tumors can be completely ablated without recurrence. This work not only provides a new strategy for regulating the photophysical properties of cyanine dyes but also establishes a versatile nanoplatform for cancer theranostics, while potentially providing new insights into the synthesis and reconstruction of other NIR dyes for different biomedical applications.

## Materials and Methods

### Materials

2,3,3-Trimethylbenzoindolenine and methyl 3-bromopropionate were purchased from Heowns (Tianjing, China). 2,2′:6′,2″-Terpyridine-4′-methanol was purchased from Bidepharm (Shanghai, China). DSPE-PEG2000 was purchased from Ponsure (Shanghai, China). Other reagents used for the synthesis of small molecules were purchased from Energy-Chemical (Shanghai, China). MB was purchased from J&K Scientific (Shanghai, China). The •OH detection probe HPF (97%) was purchased from Dalian Meilun Biotechnology Co. Ltd. (Liaoning, China). All chemicals were directly used without any further purification. Solution of Mn^2+^, Fe^2+^, Cu^2+^, and Co^2+^ ions was prepared from their chlorides.

### Preparation of LET-11, LET-11-Mn, LET-11-Fe, LET-11-Cu, and LET-11-Co

CydtPy (1 mg) was dissolved in CH_2_Cl_2_ (1 ml) under ultrasonication, followed by adding DSPE-PEG2000 (20 mg). The mixture was slowly injected into deionized water (5 ml) under stirring. The CH_2_Cl_2_ was then evaporated. The remaining aqueous solution was filtered through a polyethersulfone-syringe-driven filter (0.22 μm) (Millipore) and washed 3 times using a 30-kDa centrifugal filter under centrifugation at 3,500 rpm for 15 min at 4 °C. For LET-11-Mn, LET-11-Fe, LET-11-Cu, and LET-11-Co preparation, the method was same as the above, but an aqueous solution of MnCl_2_, FeCl_2_, CuCl_2_, or CoCl_2_ (10 μl, 4 eq) needs to be added as the initial. The concentration of LET-11, LET-11-Mn, LET-11-Fe, and LET-11-Cu solution was determined by ultraviolet-visible (UV-vis)-NIR absorption spectra according to its absorption coefficient. The resulting solutions were stored in the dark at 4 °C for further use.

### Evaluation of Fenton reaction of LET-11-Fe

LET-11-Fe solution (25 μM) was added to MB aqueous solution (2 ml, 20 μM), and then hydrogen peroxide was added. The mixture solution was irradiated under 808-nm laser (0.3 W cm^−2^) for 10 min. The absorption of MB at 665 nm was monitored using a UV-vis spectrophotometer. For ESR analysis, LET-11-Fe solution (100 μM) was added to deionized water (0.5 ml), and then hydrogen peroxide (10 μl, 30%) was added. The mixture was irradiated under 808-nm laser (0.3 W cm^−2^ ) for 10 min. The •OH trapping agent, DMPO, was added 30 min before the test in ice water.

### In vitro assays of cytotoxicity and therapy

4T1 cells were cultured in Dulbecco’s modified Eagle’s medium (DMEM) containing 10% (v/v) fetal bovine serum and 1% penicillin–streptomycin at 37°C in 5% CO_2_. 4T1 cells (7 × 10^3^ cells per well) were seeded in 96-well plates and incubated overnight at 37 °C in a humidified 5% CO_2_ atmosphere. Then, the cells were incubated with various concentrations of LET-11 or LET-11-Fe (0, 5, 10, 20, and 40 μM) for 4 h. For the in vitro cytotoxicity study, DMEM was removed from the 96-well plates following incubation for 24 h. The cells viability was measured using MTT assay. For the in vitro therapy assay, the cells were exposed to 808-nm NIR laser irradiation (0.3 or 0.5 W cm^−2^ for 10 min) after being incubated with LET-11 or LET-11-Fe for 4 h. The cells were then incubated for further 24 h. The cells viability was measured by MTT assay.

### Intracellular •OH detection

4T1 cells were seeded into confocal cell culture dishes (5 × 10^4^ cells per dish). After 24-h incubation, cells were treated with LET-11 (30 μM) or LET-11-Fe (30 μM) for 4 h. For “+ mannitol” group, cells were cocultured with mannitol (50 mM). For “+ laser” group, cells were irradiated with 808-nm laser irradiation (0.3 W cm^−2^) for 10 min at 4-h postincubation of LET-11 or LET-11-Fe. After that, the cells were incubated with HPF at 37 °C for 30 min. The cells were washed 3 times with phosphate-buffered saline, followed by fixed with 4% formaldehyde for 20 min. Finally, the 4T1 cells were observed by confocal microscopy (LSM880, Carl Zeiss, Germany).

### Flow cytometry for synergistic PTT/CDT-induced 4T1 cell death

LET-11 (30 μM) or LET-11-Fe (30 μM) solution was dispersed in DMEM cell culture. These solutions were incubated with 4T1 cells at 37 °C for 4 h. Then, trypsin treatment of cells can obtain suspension cells. The cells are divided into 4 parts (200 μl per part). Suspension cells at a density of 5 × 10^5^ cells ml^−1^ in DMEM cell culture were irradiated with 808 nm (0.3 W cm^−2^) for 10 min. The 4T1 cells were centrifuged at 1,500 rpm for 5 min. Then, the solution stained with the annexin V-fluorescein isothiocyanate (250 ng ml^−1^) and PI (250 ng ml^−1^) at 25 °C for 15 min before flow cytometry analysis (CytoFLEX, Beckman Coulter).

### Cell migration assays

4T1 cells were cultured in 96-well Cell Carrier microplate (PerkinElmer) (4 × 10^3^ cells per well) overnight at 37 °C in a humidified 5% CO_2_ atmosphere. LET-11 (30 μM) or LET-11-Fe (30 μM) was incubated with 4T1 cells for 4 h. After laser irradiation (808 nm, 0.3 W cm^−2^ for 10 min), the cell migration, speed, displacement, and roundness were recorded under digital phase contrast model using an Operetta CLS High-Content Imaging System (Operetta, PerkinElmer, USA), which quipped with a temperature and CO_2_ control option set to 37 °C and 5% CO_2_. Images were acquired for up to 12 h and recorded every 1 h. All data were processed through Harmony 4.8 Software.

### Tumor model on mice establishment

Female BALB/C nude mice aged 4 to 5 weeks were purchased from Guangdong Medicinal Laboratory Animal Center (Guangzhou, China), and All animal experiments were carried out in strict accordance with the regulations of the Animal Ethical and Welfare Committee of Shenzhen University (maximal tumor size, <2,000 mm^3^). All of the experimental mice were housed under standard conditions (temperature, ~22 °C; humidity, 40% to 70%; 12-h dark-light cycles) with free access to sterile food and water. For the establishment of xenografted tumor models, the 4T1 cells (1.5 × 10^6^ per well) were subcutaneous inoculated at the right back of mice.

### In vivo NIRFI of tumor

4T1-tumor-bearing nude mice were intravenously administrated with the LET-11 or LET-11-Mn (2 mg kg^−1^) (*n* = 3). The tumors were imaged at different time points using the InGaAs array detector (Princeton Instruments, NIRvana 640, USA) (excitation, 808 nm; 1,000-nm long-pass emission filter).

### In vivo MRI of tumor

4T1-tumor-bearing nude mice (*n* = 3) were intravenously administrated with the LET-11-Mn (2 mg kg^−1^). The *T*_1_-weighted MR images were obtained by MRI system (UMR 770 3.0T, United-Imaging, China) using the following parameters: repetition time/echo time = 550/14.8 ms, field of view = 50 × 48, and acquisition time = 25 min.

### In vivo PAI of tumor

4T1-tumor-bearing mice (*n* = 3) were intravenously administrated with LET-11-Fe (2 mg kg^−1^). PA signals at 835 nm of the tumor sites were recorded at different time points by Vevo LAZR-X system (VisualSonics Inc. NY, USA).

### In vivo antitumor efficacy

When the tumor volume reached ~100 mm^3^, antitumor efficacy of the LET-11-Fe was studied. The mice were randomly divided into 6 groups (*n* = 5) and treated with (a) saline, (b) LET-11, (c) LET-11-Fe, (d) LET-11 + L1 (0.8 W cm^−2^), (e) LET-11-Fe+L2 (0.4 W cm^−2^), and (f) LET-11-Fe + L1 (0.8 W cm^−2^) via intravenous administrations (2 mg kg^−1^). The temperature of laser groups was collected by thermal imaging camera FLIR SC300, Arlington. The body weight and tumor volume of the mice were measured every 2 d. After 15 d of treatments, all the mice were sacrificed and the weight of excised tumors was measured. The tumors and major organs were histopathologically analyzed by H&E, Ki67, and TUNEL staining.

### Statistical analysis

The data were expressed as means ± SD. Statistical calculations of experimental data were conducted using the 2-sided Student’s *t* test. The data were classified with *P* values and denoted by *P < 0.05, **P < 0.01, ***P <0.001, and ****P <0.0001.

## Data Availability

Data supporting the findings of this study can be obtained from the corresponding author upon request.

## Supplementary Materials

Supplementary Materials Experimental Procedures. Fig. S1. Synthesis routes of CydtPy. Fig. S2. ^1^H NMR spectrum of Z-2 (500 MHz, DMSO-*d*_6_). Fig. S3. MS spectrum of Z-2. Fig. S4. ^1^H NMR spectrum of Z-3 (500 MHz, CDCl_3_). Fig. S5. MS spectrum of Z-3. Fig. S6. ^1^H NMR spectrum of the CydtPy (500 MHz, CDCl_3_). Fig. S7. ^13^C NMR spectrum of the CydtPy (100 MHz, CDCl_3_/DMSO-*d*_6_). Fig. S8. MS spectrum of the CydtPy. Fig. S9. UV-vis-NIR absorption spectra (A) and fluorescence spectra (B) of CydtPy, CydtPy:Co^2+^, and CydtPy:Cu^2+^ in DMSO, respectively (excitation, 835 nm). Fig. S10. DLS and TEM images of (A) LET-11-Co and (B) LET-11-Cu. Fig. S11. The size stability of LET-11, LET-11-Fe, and LET-11-Mn in deionized water at 4 °C for 1 week (*n* = 3). Fig. S12. Fluorescence spectra of LET-11 and various metal-ion-chelated LET-11 solutions. Fig. S13. The PCE of (A) LET-11, (B) LET-11-Fe, (C) LET-11-Mn, (D) LET-11-Co, and (E) LET-11-Cu, respectively. Fig. S14. Cyclic photothermal heating and cooling of LET-11-Co and LET-11-Cu solution (808 nm, 0.3 W cm^−2^). Fig. S15. Photostability of LET-11-Co and LET-11-Cu under laser irradiation (808 nm, 1 W cm^−2^). Fig. S16. MB degradation by Fenton-like reaction from different metal-ion-chelated LET-11 with or without laser irradiation. Fig. S17. DLS (A) and TEM (B) of LET-11-Fe solution before and after laser irradiation (scale bar, 200 nm). Fig. S18. Confocal laser scanning microscopy (CLSM) images of 4T1 cells stained with calcein-acetoxymethyl ester (green)/PI (red). Groups I to V represent LET-11, LET-11-Fe, LET-11 + laser, and LET-11-Fe + mannitol + laser, respectively (scale bar, 50 μm). Fig. S19. Fluorescence images of ex vivo organs and tumors at 48-h postinjection of (A) LET-11 and (B) LET-11-Mn. (C) The corresponding fluorescence intensities of (A) and (B) (excitation: 808 nm; 1,000-nm long-pass emission filter). Fig. S20. Body weight of mice after different treatments. Fig. S21. Blood biochemistry analysis of mice after intravenous injection of saline or LET-11-Fe for 15 d. Fig. S22. H&E-stained images of dissected major organs (heart, liver, spleen, lung, and kidney) of 4T1-tumor-bearing mice after various treatments. Scale bar, 50 μm. These representative images showed no noticeable sign of organ damage. (L1 indicates 0.8 W cm^−2^ and L2 indicates 0.4 W cm^−2^; scale bar, 50 μm).
