# Supplementary material for: Terpyridine-Grafted Nitrogen-Terminal Endowing Cyanine with Metal-Ion-Regulated Photophysical Properties for Cancer Theranostics
Source: Research (Wash D C). 2023 Feb 27;6:0061. doi: 10.34133/research.0061 (PMC10013959; doi:10.34133/research.0061)
Supplement: Supplementary Materials — Experimental Procedures. Fig. S1. Synthesis routes of CydtPy. Fig. S2. 1H NMR spectrum of Z-2 (500 MHz, DMSO-d6). Fig. S3. MS spectrum of Z-2. Fig. S4. 1H NMR spectrum of Z-3 (500 MHz, CDCl3). Fig. S5. MS spectrum of Z-3. Fig. S6. 1H NMR spectrum of the CydtPy (500 MHz, CDCl3). Fig. S7. 13C NMR spectrum of the CydtPy (100 MHz, CDCl3/DMSO-d6). Fig. S8. MS spectrum of the CydtPy. Fig. S9. UV-vis-NIR absorption spectra (A) and fluorescence spectra (B) of CydtPy, CydtPy:Co2+, and CydtPy:Cu2+ in DMSO, respectively (excitation, 835 nm). Fig. S10. DLS and TEM images of (A) LET-11-Co and (B) LET-11-Cu. Fig. S11. The size stability of LET-11, LET-11-Fe, and LET-11-Mn in deionized water at 4 °C for 1 week (n = 3). Fig. S12. Fluorescence spectra of LET-11 and various metal-ion-chelated LET-11 solutions. Fig. S13. The PCE of (A) LET-11, (B) LET-11-Fe, (C) LET-11-Mn, (D) LET-11-Co, and (E) LET-11-Cu, respectively. Fig. S14. Cyclic photothermal heating and cooling of LET-11-Co and LET-11-Cu solution (808 nm, 0.3 W cm−2). Fig. S15. Photostability of LET-11-Co and LET-11-Cu under laser irradiation (808 nm, 1 W cm−2). Fig. S16. MB degradation by Fenton-like reaction from different metal-ion-chelated LET-11 with or without laser irradiation. Fig. S17. DLS (A) and TEM (B) of LET-11-Fe solution before and after laser irradiation (scale bar, 200 nm). Fig. S18. Confocal laser scanning microscopy (CLSM) images of 4T1 cells stained with calcein-acetoxymethyl ester (green)/PI (red). Groups I to V represent LET-11, LET-11-Fe, LET-11 + laser, and LET-11-Fe + mannitol + laser, respectively (scale bar, 50 μm). Fig. S19. Fluorescence images of ex vivo organs and tumors at 48-h postinjection of (A) LET-11 and (B) LET-11-Mn. (C) The corresponding fluorescence intensities of (A) and (B) (excitation: 808 nm; 1,000-nm long-pass emission filter). Fig. S20. Body weight of mice after different treatments. Fig. S21. Blood biochemistry analysis of mice after intravenous injection of saline or LET-11-Fe fo [file research.0061.f1.docx]

Terpyridine Grafted Nitrogen Terminal Endowing Cyanine with Metal Ions-Regulated Photophysical Properties for Cancer Theranostics

*Junfei Zhu, Gang He, Peng-Hang Chen, Yajie Zhang, Yafei Zhang, Shan Lei, Yu Zhang, Meng Li,* *Peng Huang,* Jing Lin**

Marshall Laboratory of Biomedical Engineering, International Cancer Center, Laboratory of Evolutionary Theranostics (LET), Guangdong Key Laboratory for Biomedical Measurements and Ultrasound Imaging, School of Biomedical Engineering, Shenzhen University Medical School
Shenzhen, 518060 (China)
E-mail: jingl@szu.edu.cn; peng.huang@szu.edu.cn

Experimental Procedures

**Characterizations**. The nuclear magnetic resonance (NMR) spectra were recorded with a Bruker AV 500 NMR spectrometer. The high-resolution mass spectra (HRMS) were recorded with ESI positive mode. Transmission electron microscopy (TEM) images were collected by a JEM-F200, Japan. Dynamic light scattering (DLS) was measured by Zeta Sizer Nano ZS90, Britain. Thermal images were measured with Arlington SC300. UV-vis absorption spectra were measured on a Cary 60 UV-vis spectrophotometer (Agilent Technology, America). The fluorescence spectra were collected by an Edinburgh Instruments F-980 fluorescence spectrometer.

Photoacoustic imaging of mice was performed by Vevo LAZR-X system (VisualSonics Inc. New York, America). Fluorescence imaging of mice were collected by InGaAs array detector (Princeton Instruments, NIRvana 640, America). *T*_1_-weighted axial MRI images of the mice was measured by MRI system (UMR 770 3.0T, United-Imaging, China). Electron spin resonance (ESR) data were obtained using a Bruker EMXplus-10/12 (Bruker, Germany).

**Synthesis of compound Z-1**. The 2,3,3-Trimethylbenzoindolenine (5 g, 23.9 mmol) and Methyl 3-bromopropionate (11.9 g, 71.8 mmol) were dissolved in toluene (50 mL). The mixture was stirred at 110 °C for 20 h and then cooled to room temperature. The precipitate was filtered and washed with ether to get the crud product Z-1 without further purification as a purple solid (6.3 g).

**Synthesis of compound Z-2**. To a methanol solution (30 mL) of compound Z-1 (6.3 g) was added 2 M NaOH solution (30 mL), then the resulting mixture was stirred at 50 °C for 3 h. After cooling to room temperature, the solvent was removed. The precipitation was

collected by filtration and washed with water, then purified by flash chromatography with gradient elution (petroleum ether/ethyl acetate of 3: 1 to CH_2_Cl_2_/methanol of 10: 1) to afford compound Z-2 as a pale-yellow solid (3.2 g, 48%). ^1^H NMR (500 MHz, DMSO-*d*_6_): 7.94 (d, *J* = 8.5 Hz, 1H), 7.79 (d, *J* = 8.1 Hz, 1H), 7.74 (d, *J* = 8.8 Hz, 1H), 7.39 (td, *J* = 6.8 Hz, 0.92 Hz, 1H), 7.21 (d, *J* = 8.8 Hz, 1H), 7.17 (t, *J* = 7.8 Hz, 1H), 4.01 (dd, *J* = 13.0 Hz, 2.0 Hz, 2H), 3.89 (t, *J* = 7.0 Hz, 2H), 3.17 (s, 1H), 2.53 (s, 1H), 1.57 (s, 6H). ESI-MS m/z: C_18_H_20_NO_2_^+^ calculated: 282.1489, found: 282.1481.

**Synthesis of compound Z-3**. To a solution of compound Z-2 (1.8 g, 6.4 mmol) in dry CH_2_Cl_2_ (70 mL) was added 1-(3-Dimethylaminopropyl)-3-ethylcarbodiimide hydrochloride (EDCl, 1.4 g, 7.6 mmol) and 4-(dimethylamino)pyridine (DMAP, 1.0 g, 7.7 mmol) at 0 °C under N_2_ atmosphere. After stirring for 30 min, 2,2':6',2''-Terpyridine-4'-methanol (1.0 g, 3.8 mmol) was added to the mixture reaction. The reaction was continued overnight at room temperature, and the solvent was removed. The residue purified by flash chromatography with gradient elution (petroleum ether/ethyl acetate = 6: 1) to afford compound Z-3 as a pale-yellow solid (560 mg, 28%). ^1^H NMR (500 MHz, CDCl_3_): 8.68 (d, *J* = 4.4 Hz, 2H), 8.58 (d, *J* = 8.0 Hz, 2H), 8.40 (s, 2H), 7.90 (d, *J* = 8.6 Hz, 1H), 7.85 (td, *J* = 7.8 Hz, 1.7 Hz, 2H), 7.71 (d, *J* = 8.2 Hz, 1H), 7.65 (d, *J* = 8.7 Hz, 1H), 7.32-7.37 (m, 3H), 7.16 (t, *J* = 7.6 Hz, 1H), 7.06 (d, *J* =8.7 Hz, 1H), 5.26 (s, 2H), 4.00-4.03 (m, 4H), 2.83 (t, *J* = 7.3 Hz, 2H), 1.62 (s, 6H). ESI-MS m/z: C_34_H_31_N_4_O_2_^+^ calculated: 527.2442, found: 527.2447.

**Synthesis of compound CydtPy**. Compound Z-3 (210 mg, 0.4 mmol), 2-chloro-1-formyl-3-hydroxymethylene cyclohexene (34.3 mg, 0.2 mmol), and sodium acetate (49 mg, 0.3 mmol) were dissolved in acetic anhydride (10 mL). The mixture was stirred at room temperature for 4 h. The green solution was added dropwise to the ether solution. The green solid was filtered and collected. The crude product was purified by column silica gel chromatography with the eluent of dichloromethane/methanol to obtain the compound CydtPy as a dark green solid (71.4 mg, 30%). ^1^H NMR (500 MHz, CDCl_3_): 8.68 (d, *J* = 4.0 Hz, 6H), 8.38-3.41 (m, 6H), 8.05 (d, *J* = 8.5 Hz, 2H), 7.88-7.91 (m, 8H), 7.71 (dd, *J* = 5.7 Hz, 3.4 Hz, 1H), 7.54-7.60 (m, 5H), 7.45 (t, *J* = 7.6 Hz, 2H), 7.37 (t, *J* = 5.6 Hz, 4H), 6.43 (d, *J* = 14.0 Hz, 2H), 5.25 (s, 4H), 4.82 (t, *J* = 6.7 Hz, 4H), 4.31 (t, *J* = 6.7 Hz, 2H), 3.21 (t, *J* = 6.3 Hz, 4H), 2.71 (t, *J* = 5.8 Hz, 4H) 1.97 (s, 12H). ^13^C NMR (100 MHz, CDCl_3_/DMSO-*d*_6_) 178.61, 174.98, 172.29, 160.28, 159.88, 155.42, 153.61, 150.89, 148.72, 143.76, 138.43, 136.69, 135.79, 135.59, 134.91, 133.52, 132.86,, 132.34, 130.06, 129.05,126.19, 124.18, 115.50, 105.79, 70.32, 55.86, 36.97, 35.32, 34.34, 32.42, 30.98, 27.36, 25.32, 23.86. ESI-MS m/z: C_76_H_66_ClN_8_O_4_^+^ calculated: 1189.4890, found: 1189.4884; 595.2476 (M+1)^2+^; 397.1678 (M+2H)^3+^

**Photothermal effect of** **LET-11, LET-11-Mn, LET-11-Fe, LET-11-Cu and LET-11-Co**. The photothermal performance of LET-11, LET-11-Mn, LET-11-Fe, LET-11-Cu and LET-11-Co was investigated under 808 nm laser irradiation. In short, the above solutions (25 μM, 50 μL) were irradiated at 0.3 W cm^-2^ until the solution temperature reached a steady state. Then, the solution naturally cooled down to the ambient temperature after the removal of laser irradiation. The process was collected using a thermal imaging camera FLIR SC300, Arlington. For photothermal stability, the above solutions (25 μM, 50 μL) was irradiated at 0.3 W cm^-2^ for 3 min, then cooled for 3 min for four cycles.

The photothermal conversion efficiency (η) of the nanoparticles was calculated using the following equation [1]:

$$\eta=\frac{hA\left( T_{max}-T_{amb} \right)-Q_{0}}{I\left( 1-{10}^{-A_{\lambda}} \right)}$$

Where *h* is the heat transfer coefficient, *A* is the surface area of the solution, *T*_max_ and *T*_amb_ are the maximum system temperature and surrounding temperature, respectively. *Q*_0_ is the baseline energy inputted by the sample cell. *I* is the laser power and *A_λ_* is the absorbance of nanoparticles solution.

**Supplementary Figures:**

**Figure S1**. Synthesis routes of CydtPy.


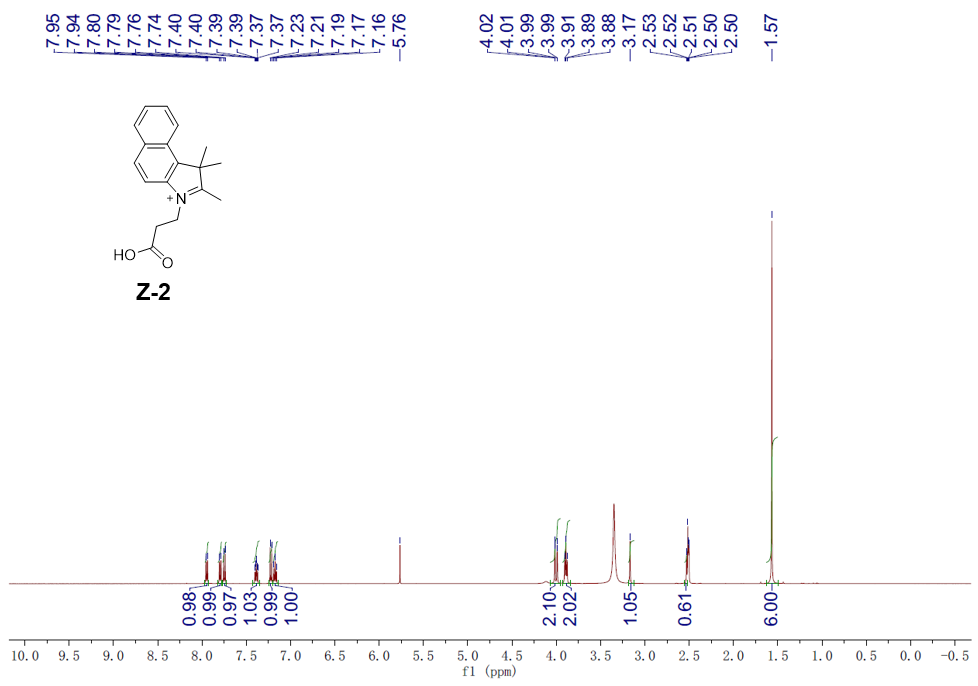


**Figure S2**. The ^1^H NMR spectrum of Z-2 (500 MHz, DMSO-*d*_6_).


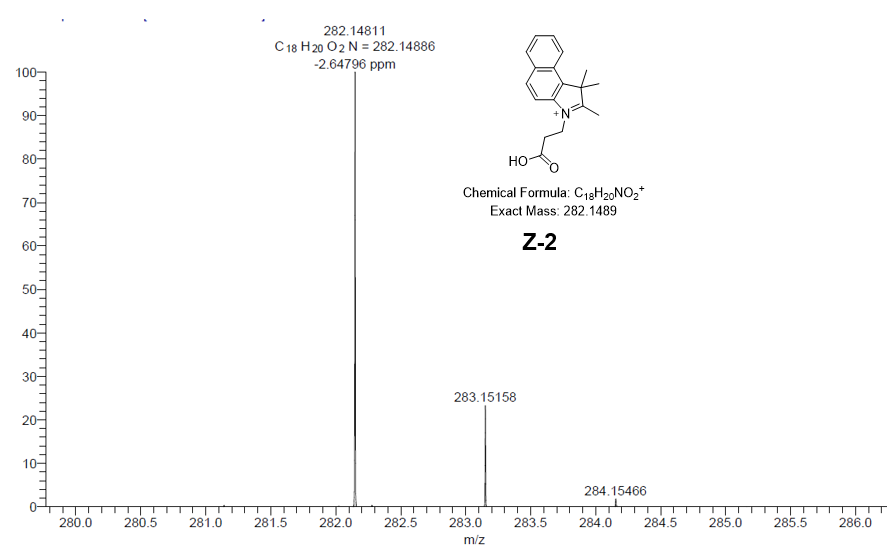


**Figure S3**. The MS spectrum of Z-2.


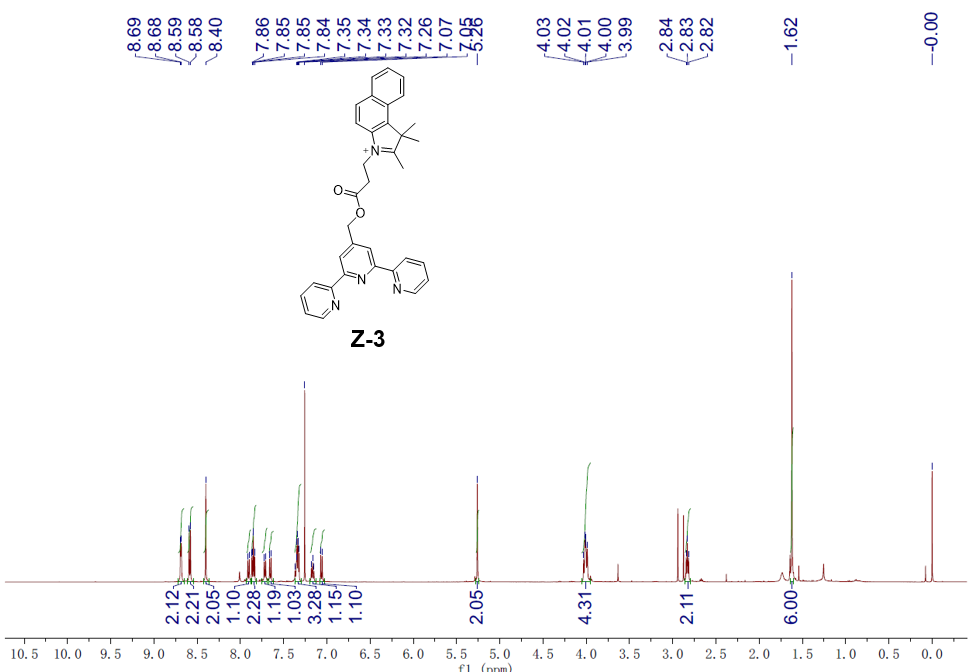


**Figure S4**. The ^1^H NMR spectrum of Z-3 (500 MHz, CDCl_3_).


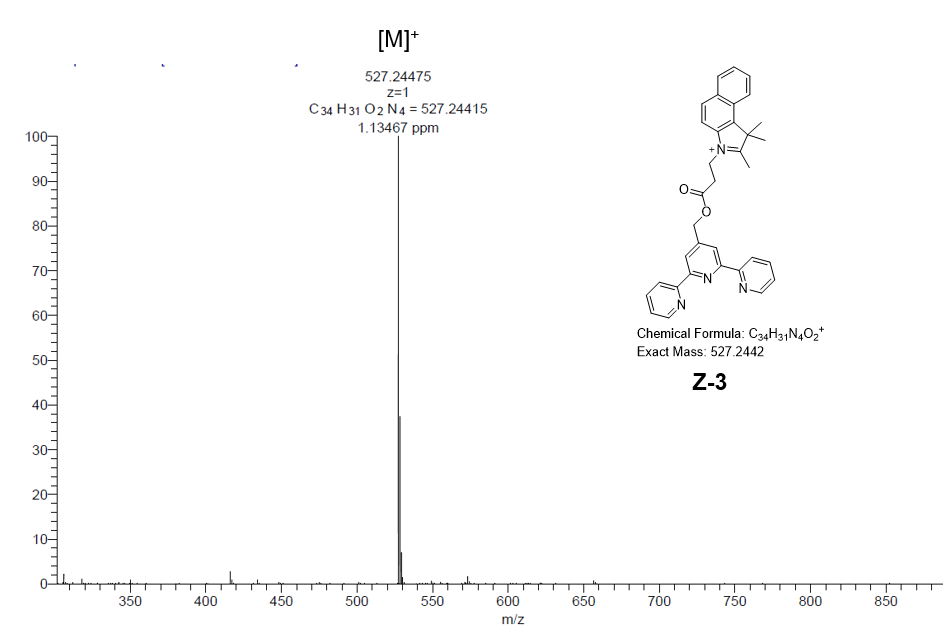


**Figure S5**. The MS spectrum of Z-3.


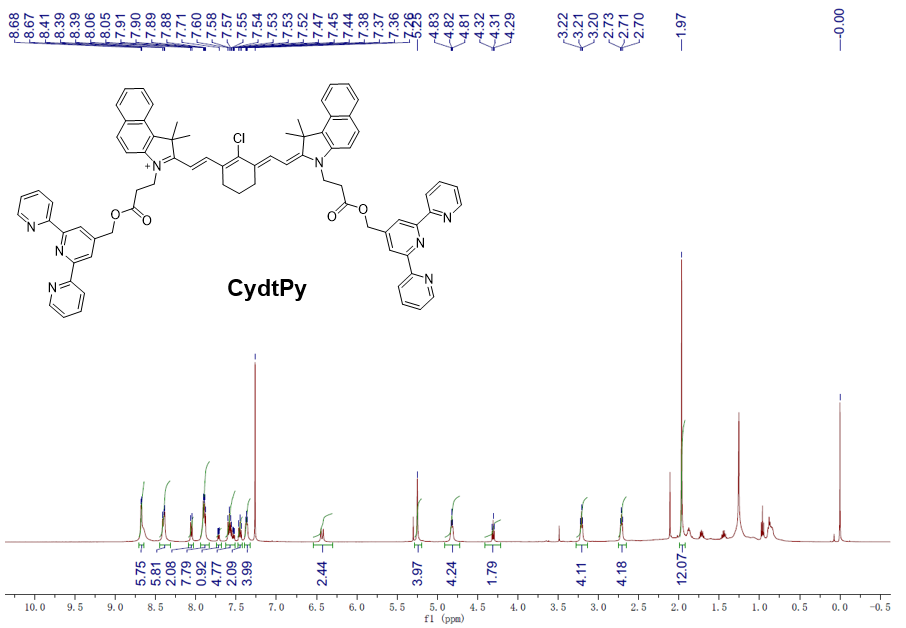


**Figure S6**. The ^1^H NMR spectrum of the CydtPy (500 MHz, CDCl_3_).


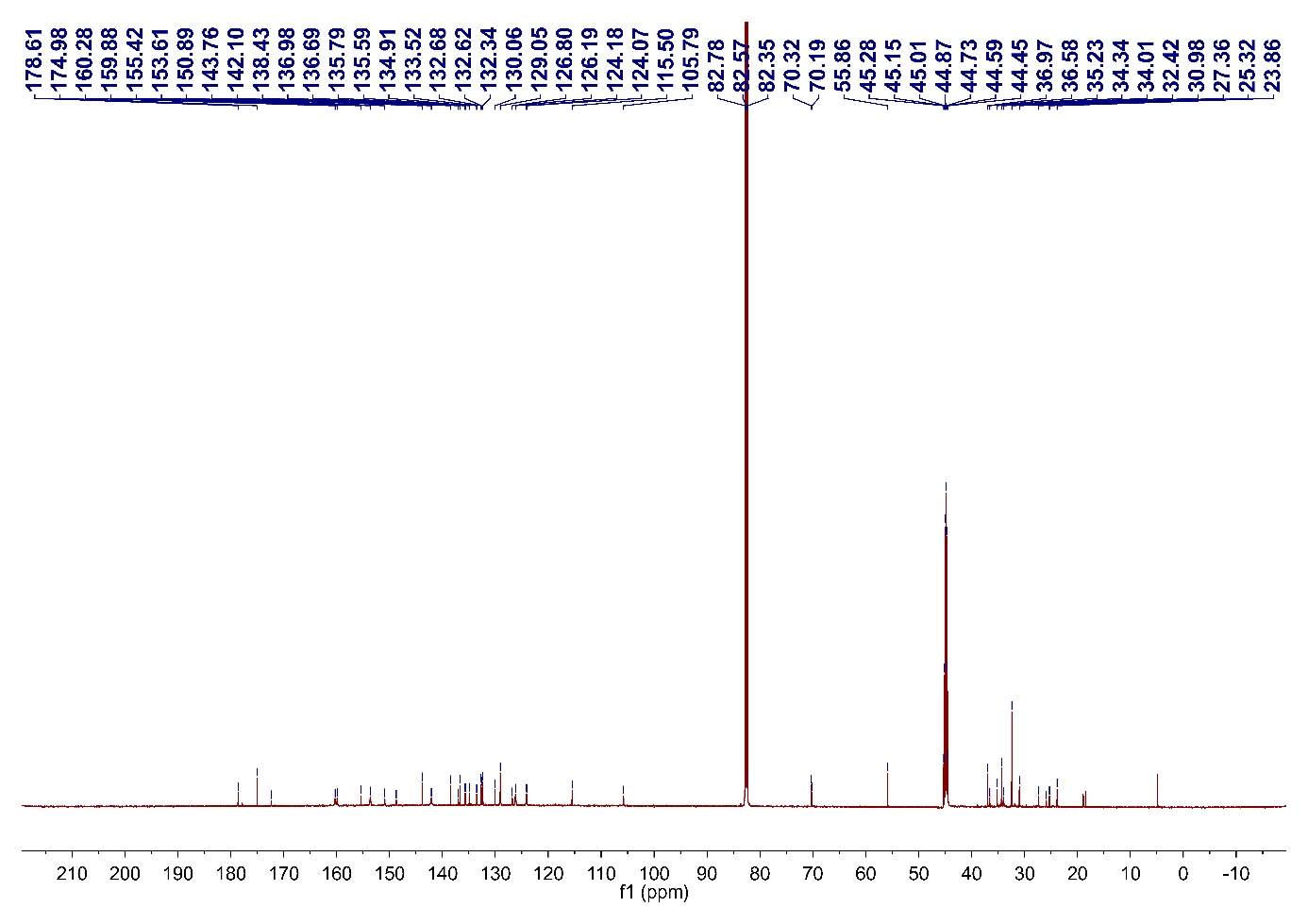


**Figure S7**. The ^13^C NMR spectrum of the CydtPy (100 MHz, CDCl_3_/DMSO-*d*_6_).


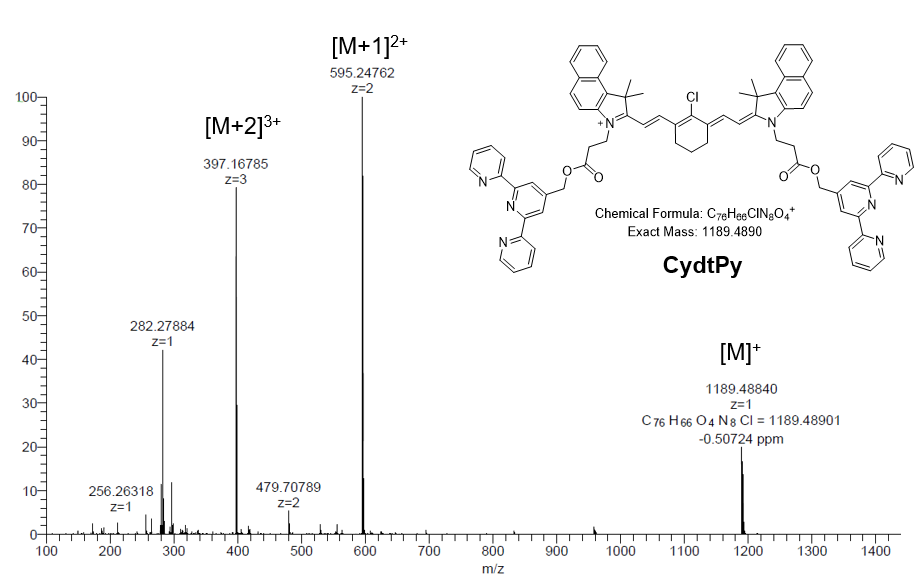


**Figure S8**. The MS spectrum of the CydtPy.


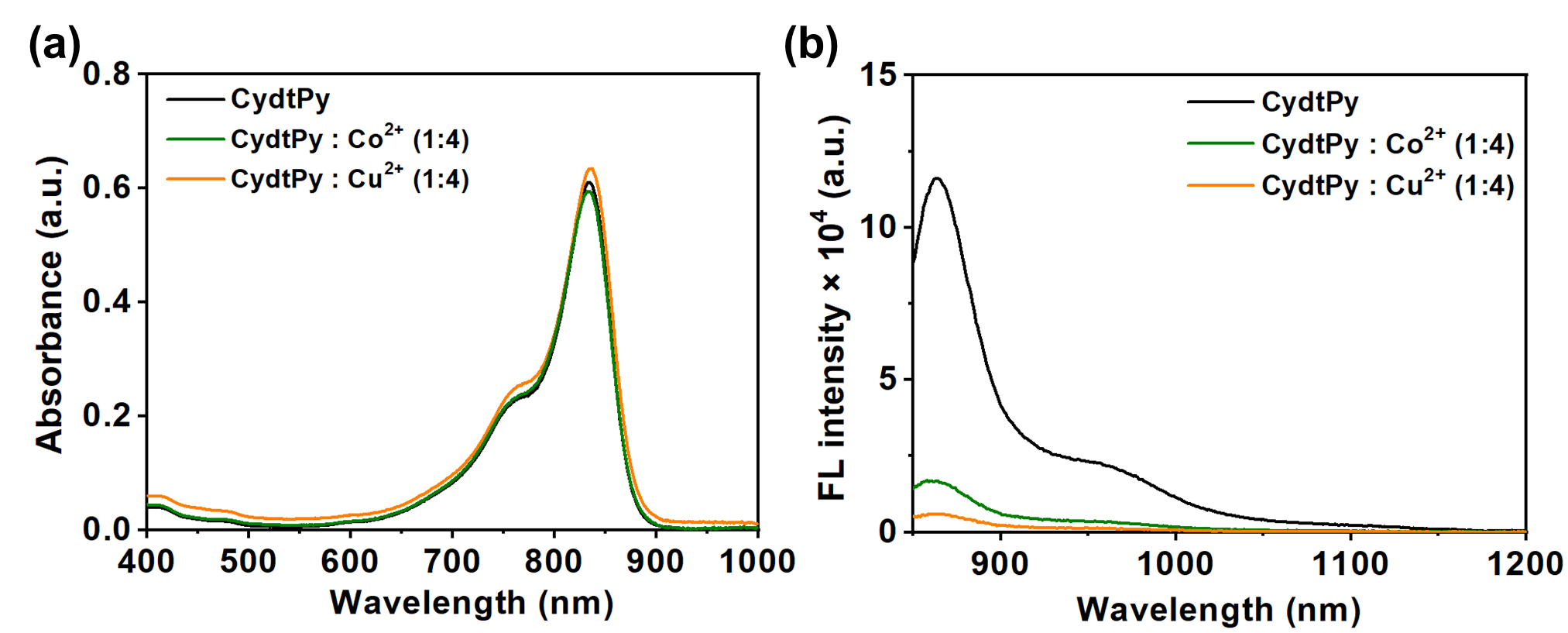


**Figure S9**. UV-vis-NIR absorption spectra (a) and fluorescence spectra (b) of CydtPy, CydtPy:Co^2+^ and CydtPy:Cu^2+^ in DMSO, respectively (Ex: 835 nm).


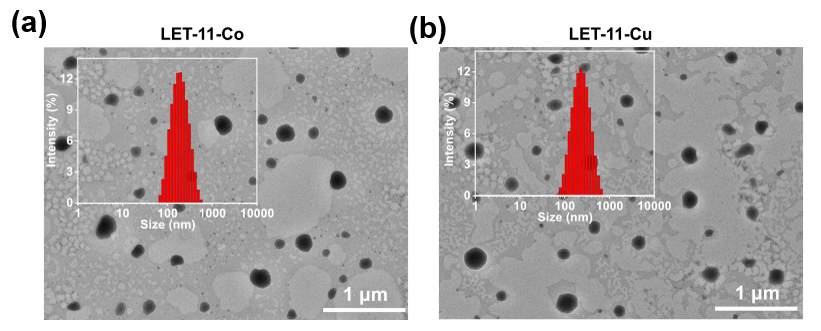


**Figure S10**. DLS and TEM images of (a) LET-11-Co and (b) LET-11-Cu.


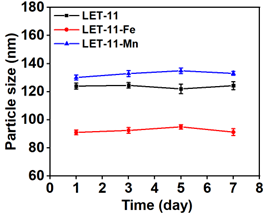


**Figure S11**. The size stability of LET-11, LET-11-Fe and LET-11-Mn in deionized water at 4 °C for one week (n = 3).


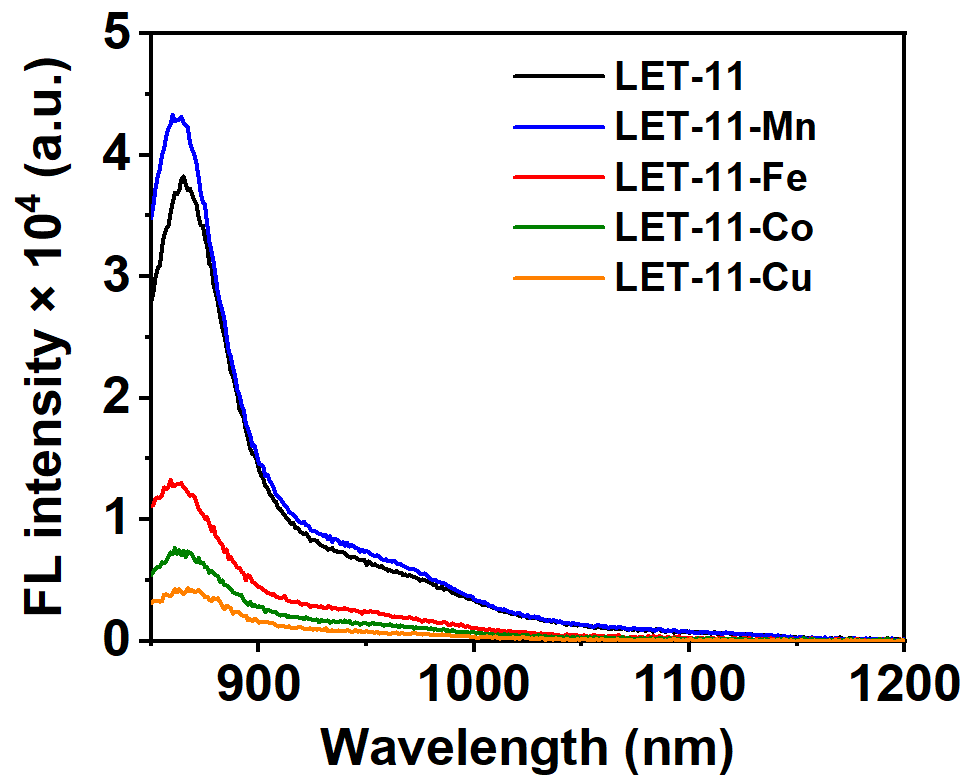


**Figure S12**. Fluorescence spectra of LET-11 and various metal ion-chelated LET-11 solutions.


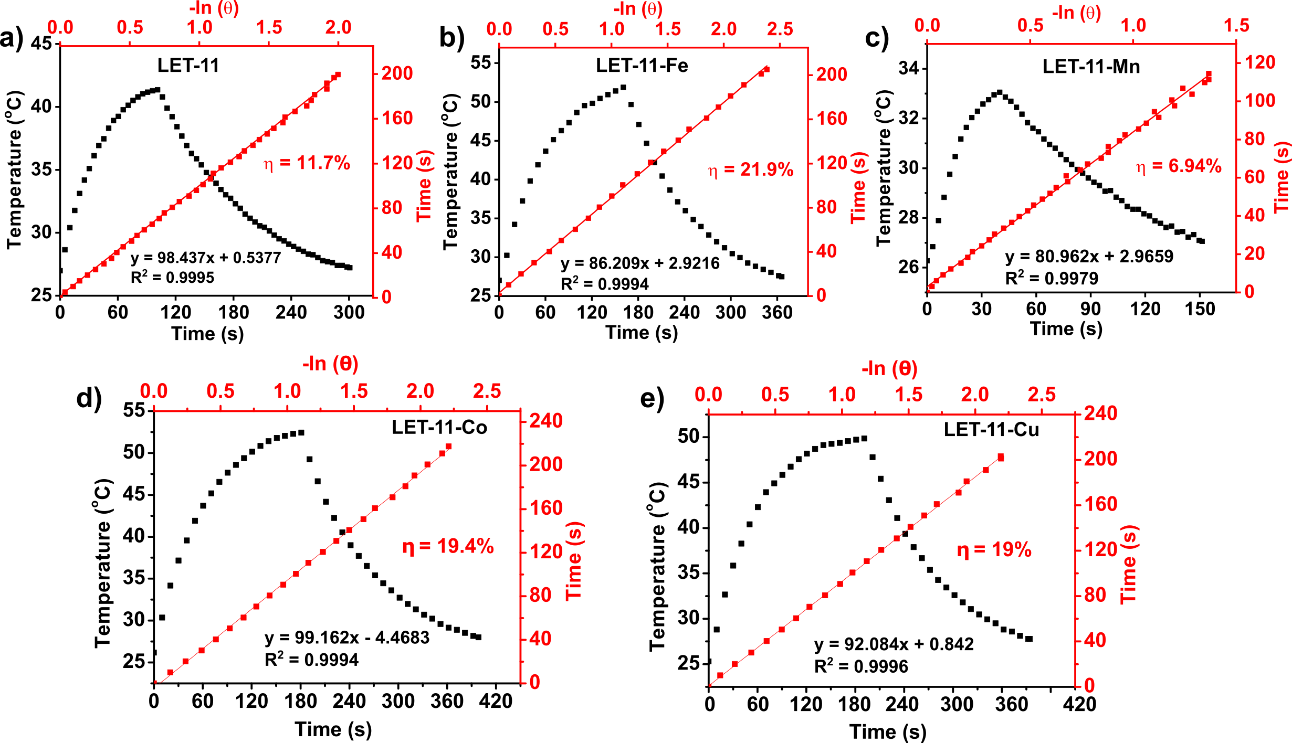


**Figure S13**. The photothermal conversion efficiency of (a) LET-11, (b) LET-11-Fe, (c) LET-11-Mn, (d) LET-11-Co and (e) LET-11-Cu, respectively.


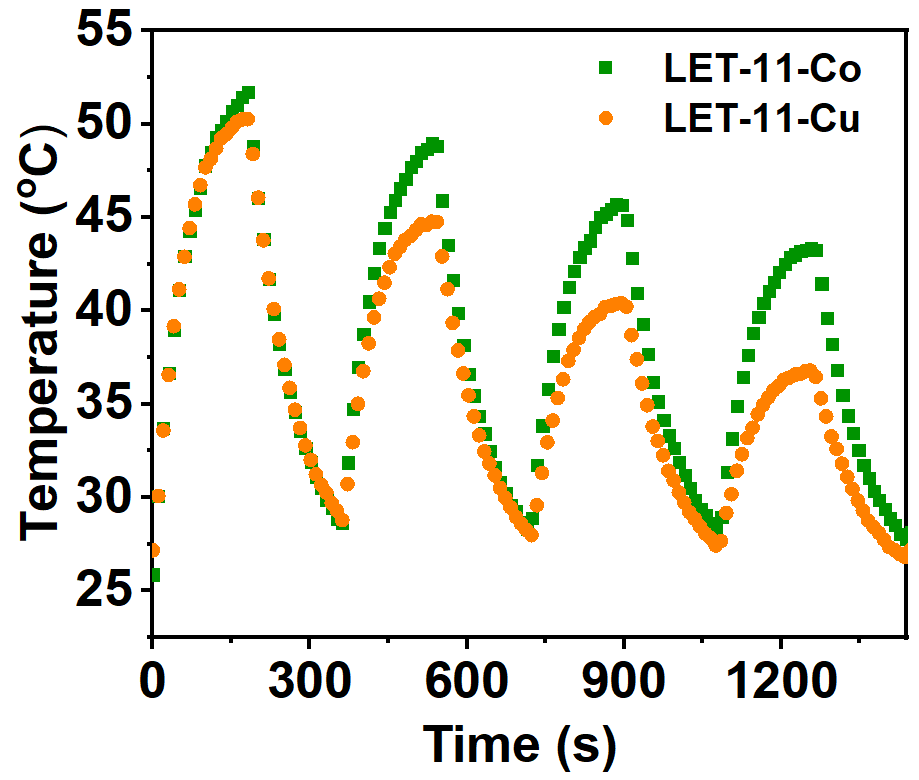


**Figure S14**. Cyclic photothermal heating and cooling of LET-11-Co and LET-11-Cu solution (808 nm, 0.3 W cm^-2^).


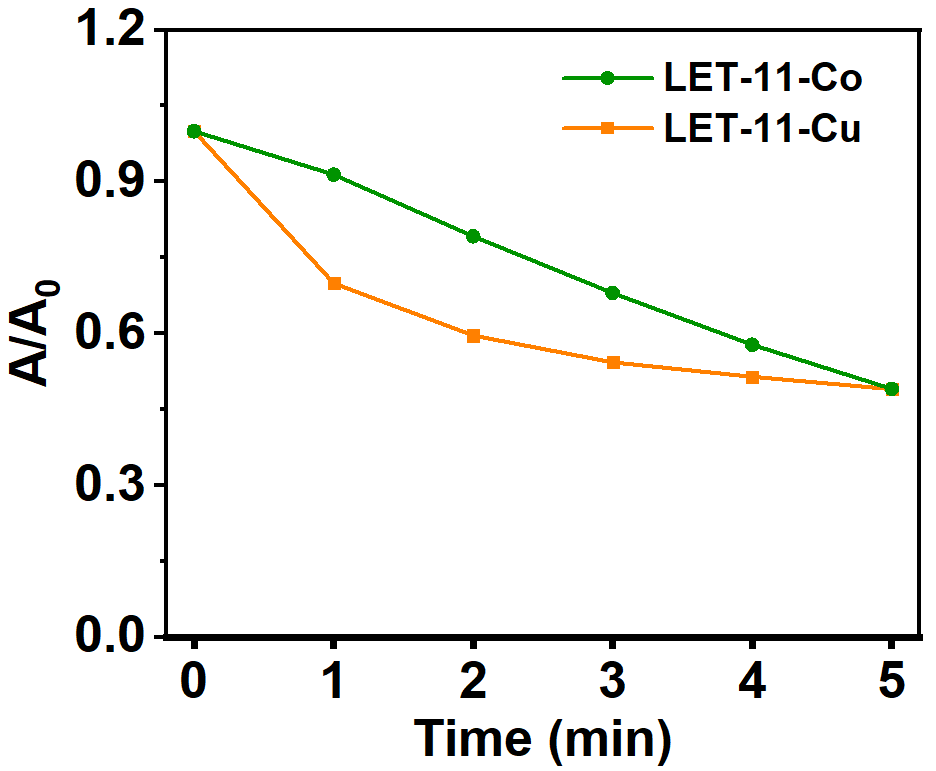


**Figure S15**. Photostability of LET-11-Co and LET-11-Cu under laser irradiation (808 nm, 1 W cm^-2^).


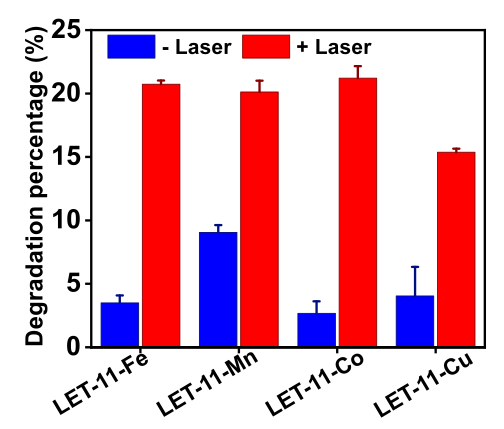


**Figure S16**. MB degradation by Fenton-like reaction from different metal ion-chelated LET-11 with or without laser irradiation.


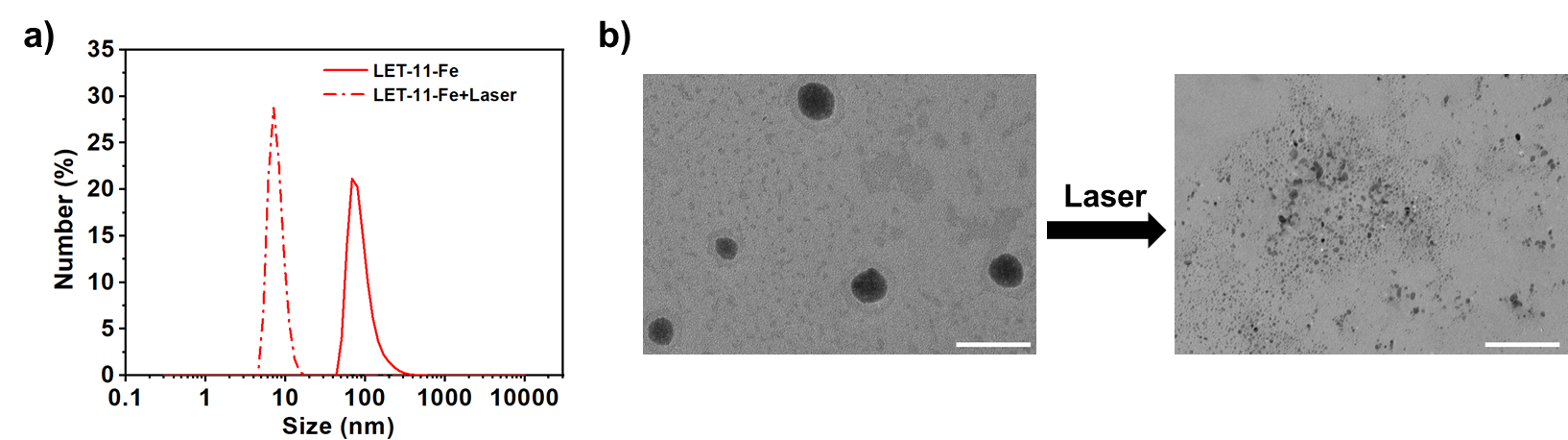


**Figure S17**. DLS (a) and TEM (b) of LET-11-Fe solution before and after laser irradiation (Scale bar = 200 nm).


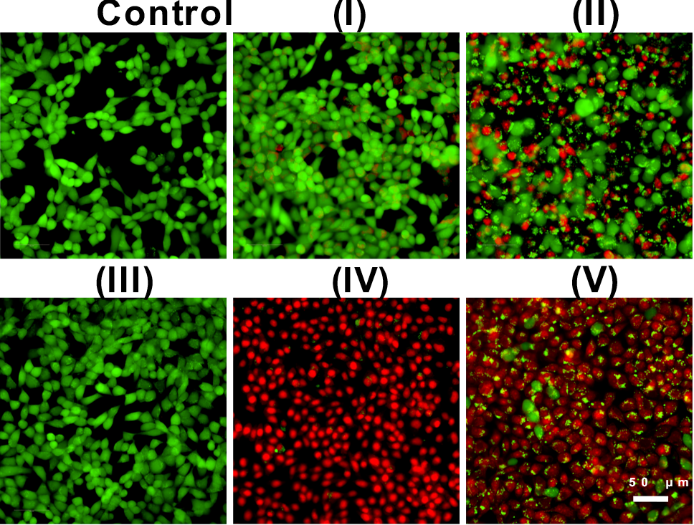


**Figure S18**. CLSM images of 4T1 cells stained with calcein AM (green)/PI (red). Groups I-V represent LET-11, LET-11-Fe, LET-11+Laser, and LET-11-Fe+mannitol+Laser, respectively (Scale bar = 50 μm).


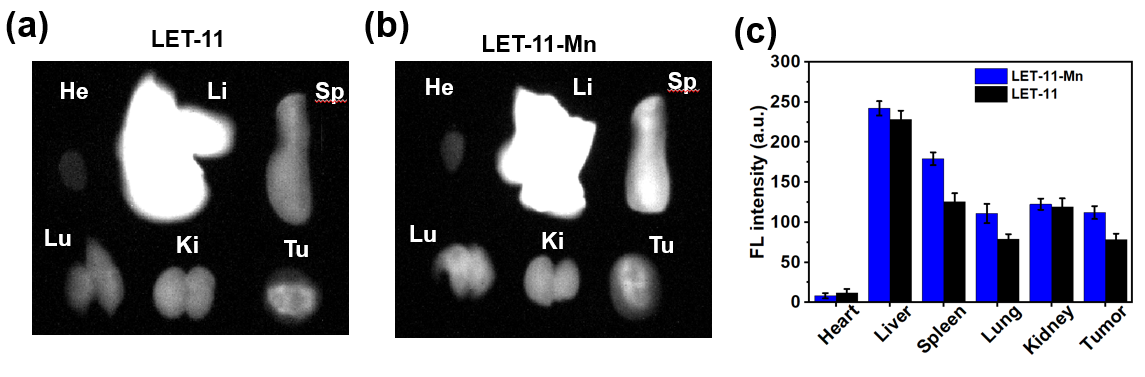


**Figure S19**. Fluorescence images of *ex vivo* organs and tumors at 48 h post-injection of (a) LET-11 and (b) LET-11-Mn. (c) The

corresponding fluorescence intensities of (a) and (b) (Ex: 808 nm, 1000 nm long-pass emission filter).


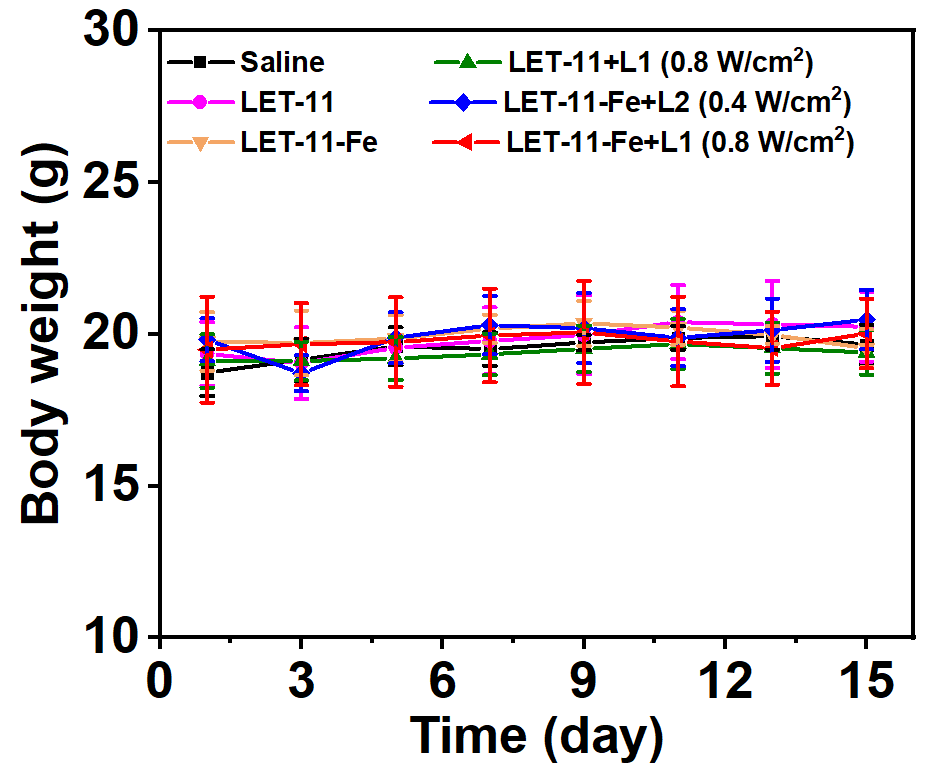


**Figure S20**. Body weight of mice after different treatments.


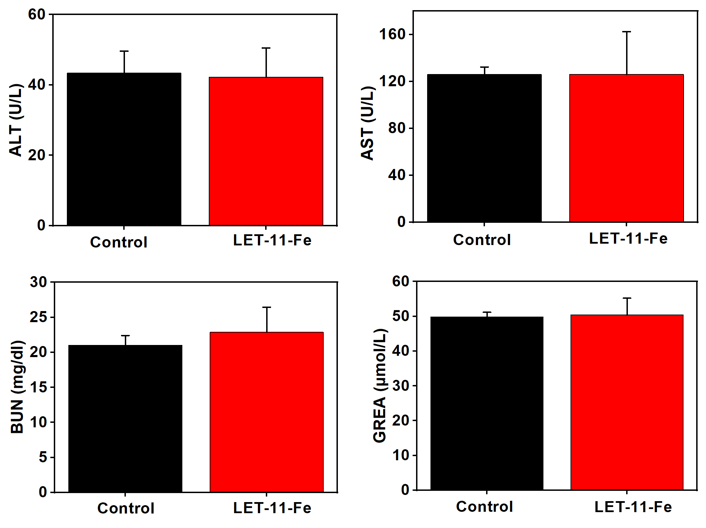


**Figure S21**. Blood biochemistry analysis of mice after intravenous injection of saline or LET-11-Fe for 15 days.


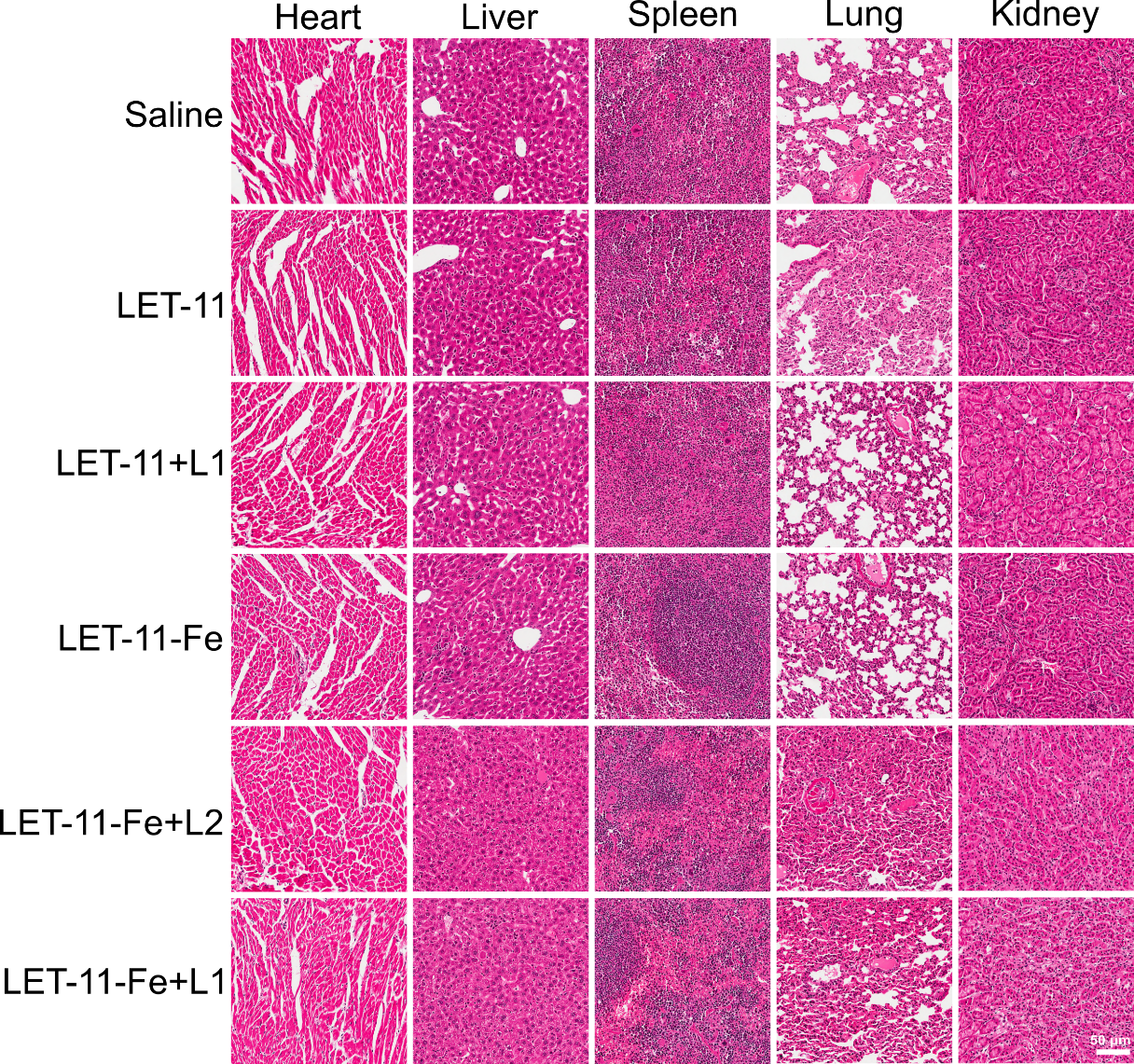


**Figure S22**. H&E stained images of dissected major organs (heart, liver, spleen, lung, kidney) of 4T1 tumor-bearing mice after various treatments. Scale bar: 50 μm. These representative images showed no noticeable sign of organ damage. (L1 indicates 0.8 W cm^−2^ and L2 indicates 0.4 W cm^−2^, Scale bar = 50 μm).

# References

[1] D. K. Roper, W. Ahn and M. Hoepfner, “Microscale heat transfer transduced by surface plasmon resonant gold nanoparticles,” *The Journal of Physical Chemistry C*, vol. 111, no. 9, pp. 3636-3641, 2007.
